# Lactate signalling leads to aggregation of immune-inflammatory hotspots and SLC5A12 blockade promotes their resolution

**DOI:** 10.1038/s42255-025-01331-9

**Published:** 2025-08-04

**Authors:** Michelangelo Certo, Elena Pontarini, Sebastian G. Gilbert, Ronny Schmidt, Jason D. Turner, Davide Lucchesi, Daria Apostolo, Giulia Cavallaro, Charlotte G. Smith, Serena Colafrancesco, Joana Campos, Saba Nayar, Christoph Schröder, Benjamin A. Fisher, Fabian Spill, Michele Bombardieri, Claudio Mauro

**Affiliations:** 1 Department of Inflammation and Ageing, School of Infection, Inflammation and Immunology, College of Medicine and Health, University of Birmingham, Birmingham, UK; 2 Experimental Medicine and Rheumatology, William Harvey Research Institute, Queen Mary University of London, London, UK; 3 School of Mathematics, University of Birmingham, Birmingham, UK; 4 Sciomics GmbH, Neckargemünd, Germany; 5 Department of Translational Medicine, University of Piemonte Orientale (UPO), Novara, Italy; 6 Department of Physics and Astronomy, University of Catania, Catania, Italy; 7 Department of Biomedical Sciences, Humanitas University, Pieve Emanuele, and Rheumatology and Clinical Immunology, Istituto di Ricovero e Cura a Carattere Scientifico, IRCCS Humanitas Research Hospital, Rozzano, Italy; 8 NIHR Birmingham Biomedical Research Centre, University Hospitals Birmingham NHS Foundation Trust and University of Birmingham, Birmingham, UK; 9 Present Address: Birmingham Tissue Analytics, Institute of Translational Medicine, University of Birmingham, Birmingham, UK; 10 Present Address: Propath UK Limited, Hereford, UK

**Keywords:** Immunology, Rheumatology, Metabolism

## Abstract

Ectopic lymphoid structures (ELS) are aggregates of lymphoid cells that often form within inflamed tissues in patients with autoimmune diseases, cancer, infectious diseases and cardiovascular conditions. These structures drive B cell maturation into memory B cells and plasma cells through B cell and T cell co-stimulation, and their role in pathogenesis is increasingly recognized. Understanding how ELS develop and persist in inflamed tissues is essential for elucidating the pathogenesis and treatment responses in diseases in which they have a prominent role. Here we show that metabolic pathways and specific metabolites, in particular lactate, are master regulators of ELS organization in Sjögren’s disease (SjD), the second-most common autoimmune rheumatic disease. Furthermore, inhibiting lactate uptake by lactate transporters, specifically by SLC5A12 blockade, represents a previously unappreciated checkpoint in autoimmune inflammatory diseases. This approach results in multidimensional pro-resolution effects, including reduced inflammatory cytokine levels, enhanced T cell egress from inflamed sites and diminished T cell and B cell areas and their segregation within ELS.

## Abstract (web summary)

Certo, Pontarini et al. provide insight into the metabolic requirements of ectopic lymphoid structure (ELS) assembly in the context of autoimmunity, and show that blocking lactate uptake by SLC5A12 offers therapeutic benefits in a mouse model of Sjögren’s disease.

## Main

ELS drive B cell maturation into plasma cells through B cell and T cell co-stimulation. Understanding how ELS develop and are maintained in inflamed tissues is crucial, as they impact the progression of infections, autoimmune diseases, cancer, atherosclerosis and transplant rejection^1^. In certain autoimmune conditions, patients with ELS in the inflamed tissue often respond poorly to standard biologics, presenting a substantial challenge therapeutically^1,2^. Conversely, intra-tumoral ELS in solid cancers have been linked to improved clinical responses to immune checkpoint inhibitors^3–5^.

Multiple autoimmune diseases, including rheumatoid arthritis, multiple sclerosis and SjD, are characterized by ELS development in target organs. SjD—the second-most common autoimmune rheumatic disease, affecting 0.2–0.5% of the general population—is an excellent model for studying ELS because of the accessibility of labial salivary gland (SG) biopsies and the availability of inducible models of sialadenitis that form ELS^6^.

Unlike other inflammatory and autoimmune diseases, in which evidence of ELS influencing disease evolution and treatment response to conventional and biologic treatments is emerging, there is robust evidence that ELS have a pathogenic role in SjD. For example, they are associated with more severe systemic manifestations, including lymphadenopathy and peripheral neuropathy, and a significantly higher risk of progression to B cell MALT lymphoma^1,7–10^.

The mechanisms regulating ectopic lymphoid neogenesis in human pathology remain poorly defined. Although these processes involve interactions between immune and non-immune cells specific to the local microenvironment where ELS form, they seem to follow stereotypical key steps essential for their development and function across different conditions^2^. Similar to lymphoid organogenesis during embryonic life, lymphotoxin b (LTb) is critically involved in ectopic lymphoid development, leading to the ectopic expression of homoeostatic chemokines such as CXC-chemokine ligand 13 (CXCL13), CC-chemokine ligand 19 (CCL19) and CCL21. This results in increased infiltration of B cells and T cells expressing their specific receptors, CXCR5 (for CXCL13) and CCR7 (for CCL19 and CCL21)^11^.

In addition to the classical model of lymphoid neogenesis described above, pro-inflammatory cytokines, particularly those related to early activation of the interleukin (IL)-22–IL-17 pathway and produced by both γδ and conventional CD4 T cells, can also initiate or propagate ELS by inducing lymphoid chemokine production^12–18^.

Within the forming ELS, CD4 T cells with B cell helper functions, such as T follicular helper cells (T_FH_) and the recently described subset of T peripheral helper cells, promote B cell differentiation into germinal centre B cells primarily by the release of the key B cell activating cytokine IL-21 and synergistic co-stimulation through interactions between ICOS–ICOSL and CD154–CD40 (refs. ^19–23^). Although CD4 T cells have a critical role in ELS formation and function, the signals regulating CD4 T cell responses within the local inflammatory microenvironment of developing ELS remain poorly understood. Recent discoveries highlighting the fundamental role of metabolism in regulating immune cell biology and inflammatory pathways have substantially advanced our understanding of immune cell regulation and related pathologies^24,25^.

Studies have recently revealed emerging signalling functions of intermediates and end products of metabolism, such as lactate, acetyl-CoA and succinate, in regulating immunity^26–29^. These metabolites influence cytokine production, interact with transcription factors, modulate ion channel activity and affect cell migration and differentiation^28^. This evolving view suggests that metabolite signalling enables cell-to-cell communication and allows sensing of microenvironmental conditions to trigger stress responses and cellular adaptation^28^.

Lactate, traditionally viewed as a by-product of metabolism or a biomarker, has recently been recognized as a multifaceted, bioactive molecule. Lactate accumulation in inflammatory sites significantly impacts tissue-resident and infiltrating immune cells as well as stromal cells. It has been linked to tumour escape from immune surveillance mechanisms through inhibition of macrophage and T cell effector functions^30–34^. At the site of inflammation, accumulation of lactate can lead to metabolic reprogramming, inflammation and angiogenesis^27,28,35,36^.

In health, lactate concentrations in blood and tissues are around 1.5–2 mM but can increase to 10–40 mM in inflamed tissues, such as atherosclerotic plaques, adipose tissue in obesity, tumour microenvironments and arthritic joints^37–39^. Elevated lactate levels have been reported in the serum of patients with multiple sclerosis and SjD, with the latter correlating with fatigue and exercise intolerance^36,40^. Lactate is produced in the cytoplasm during hypoxia or as a result of aerobic glycolysis in proliferating cells and is then secreted through the plasma membrane. This transport is mediated by six known solute carrier transporters that perform proton–lactate symport (MCT1–4) or sodium-dependent transport (SMCT1 (SLC5A8) and SMCT2 (SLC5A12)). The transport direction of both systems depends on the lactate gradient, favouring lactate import when extracellular lactate is elevated, such as in inflamed tissues^41,42^.

The lactate transporter SLC5A12 is mostly expressed in the distal tubules in the kidney in healthy conditions but has been shown to be upregulated in subsets of CD4 T helper (T_H_) cells (T_H_1, T_H_17, T_FH_) and B cells within inflamed tissues, but not in the periphery, in autoimmune conditions such as the synovium in rheumatoid arthritis^43,44^.

The critical role of CD4 T cells in ELS formation and function has been demonstrated^1,20^, and we and others have reported the profound immunoregulatory effects of lactate and its associated transporters on CD4 T cell function, leading to CD4 T cell entrapment in the inflamed tissue and upregulation of key cytokines such as IL-17 and IL-22 (refs. ^44,45^). We also previously reported that lactate feeding to activated CD4 T cells increases the intracellular pool of acetyl-CoA, initiating a cascade of fatty acid synthesis activation^44^. However, acetyl-CoA can also be used as a substrate for acetylation of various targets, including histones, enzymes and cholesterol or fatty acid synthesis, as well as transcription factors^46^.

Given the role of CD4 T cells in regulating ELS formation and function and the importance of lactate in modulating CD4 T cell functions, including migration and IL-17 and IL-22 cytokine outputs, we explored the capacity of lactate and lactate transporters to impact ELS development in the SGs of both animal models and patients with SjD.

## Results

### Lactate transporter expression associates with metabolism and ELS in SjD

**Fig. 1:**
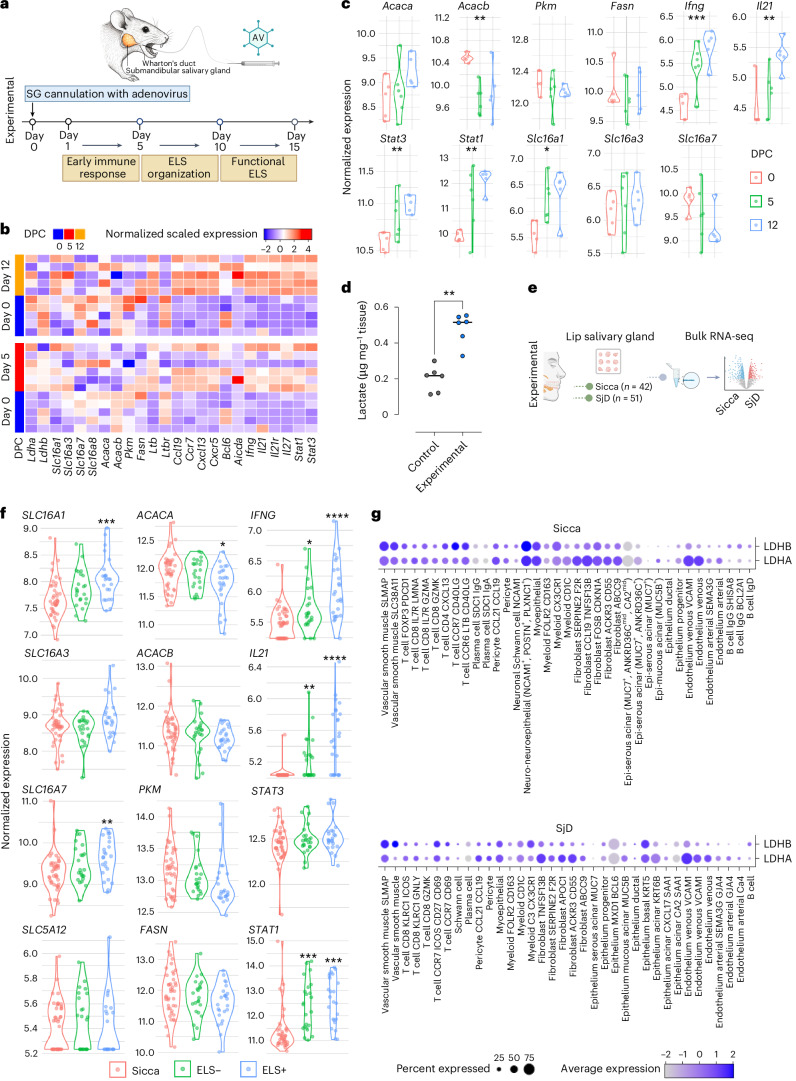
Transcriptomic analysis and lactate quantification in SG tissue. **a**, Workflow of the murine sialadenitis model, showing a representation of the mouse submandibular SG and the cannula used for the delivery of the AdV (AV) vector into the gland. The timeline depicts ELS formation within the SGs following injection of the AdV type-5 vector. **b**, Supervised heatmap of differentially expressed genes from bulk RNA-seq of murine SGs collected at 0, 5 and 12 days post cannulation (DPC). **c**, Expression levels of selected genes related to lactate transporters, metabolic enzymes downstream of lactate signalling and inflammatory mediators in murine SGs collected at 0, 5 and 12 days post cannulation (*n* = 10 per time point). Adjusted *P* values calculated using DESeq2 (*P < 0.05, **P < 0.01, ***P < 0.001, ****P < 0.0001). **d**, Lactate concentrations in SGs from non-cannulated (control) and cannulated (experimental) mice at day 5 (*n* = 6 glands per group). Data are expressed as means, normalized to tissue weight; error bars, s.d. Statistical significance was assessed using the Mann–Whitney *U*-test, **P < 0.01. **e**, Schematic overview of the bulk RNA-seq workflow used for analysing human SG tissue. **f**, Expression levels of selected mRNA transcripts related to lactate transporters, metabolic enzymes downstream of lactate signalling and inflammatory mediators in human SGs. This comparison involves sicca and SjD SGs classified as ELS− or ELS+ based on histological analysis of matched biopsies (adjusted *P* values calculated by DESeq2, *P < 0.05, **P < 0.01, ***P < 0.001, ****P < 0.0001). **g**, Scaled average expression and the percentage of cells with any expression of lactate dehydrogenase (LDHA or LDHB) mRNA transcripts per cell cluster in both SjD and non-SjD sicca SGs. Data were generated from disaggregated minor labial SG biopsies (*n* = 7 patients per disease).

Retrograde cannulation of the submandibular SGs in C57BL/6 mice is an effective method to deliver a replication-deficient adenovirus (AdV)-5, inducing the formation of ELS within the SGs^6^. Following AdV-5 infection, a stepwise infiltration of innate immune cells occurs in the gland, with an early immune response in the SGs during the first 3 days post viral cannulation and activation of key lymphomagenesis pathways by day 5. The initial organization of ELS, characterized by segregated T cell and B cell areas, occurs before day 10, with fully functional ELS development by day 10 onwards (Fig. 1a). SGs were collected at days 0, 5 and 12 post cannulation for longitudinal bulk RNA sequencing (RNA-seq) analysis.

**Extended Data Fig. 1:**
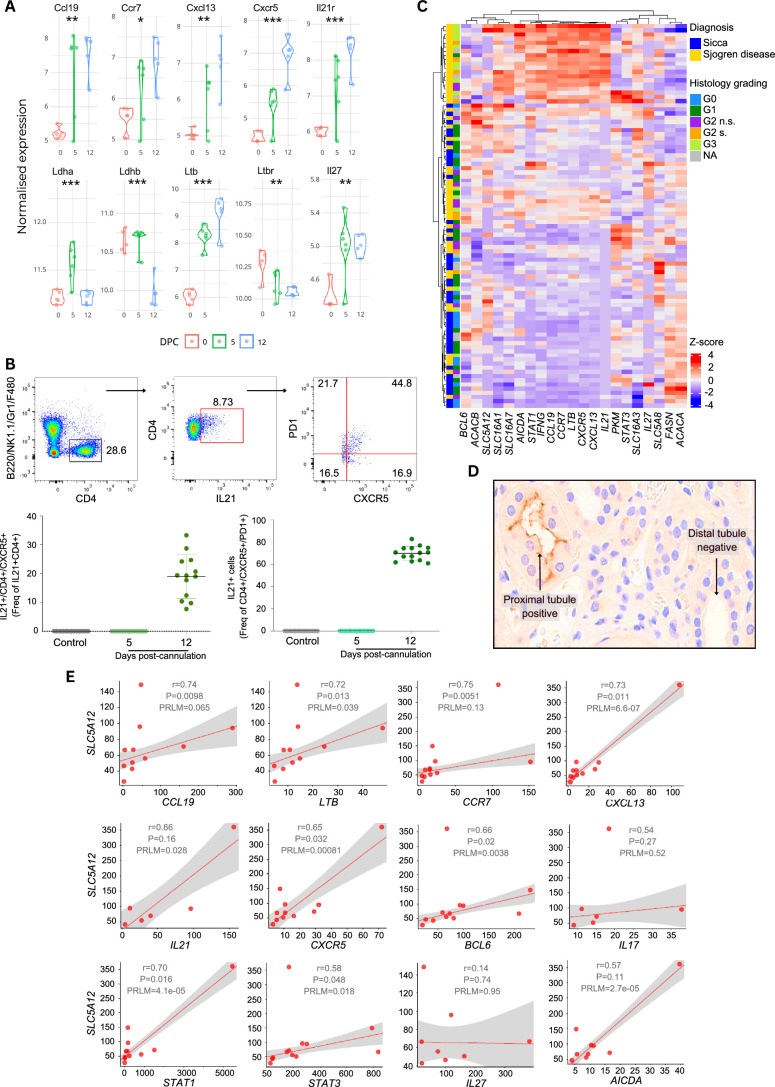
Gene expression and correlation analysis of inflammatory mediators in salivary glands. **(a)** Expression levels of selected genes related to inflammatory mediators involved in ectopic lymphoid structure (ELS) formation and function in murine salivary glands collected at 0-, 5-, and 12-days post-cannulation. Adjusted p-values calculated by DESeq2 (*p < 0.05, **p < 0.01, ***p < 0.001, ****p < 0.0001). **(b)** Flow cytometry gating strategy showing the phenotype of CD4^+^ T cells producing IL21 (top panel). Frequency of IL21-producing T CD4+ cells with a Tfh-phenotype (CD4 + / CXCR5 + , bottom left panel), and frequency of IL21+ cells in the Tfh cell population (CD4 + / CXCR5 + /PD1 + , bottom right panel) in murine salivary glands collected at 0-, 5-, and 12-days post-cannulation.Data are expressed as mean ± SD from n = 13 glands per group. **(c)** Unsupervised heatmap of differentially expressed genes (DEGs) comparing SjD patients versus sicca controls. **(d)** Immunohistochemistry staining of human kidney tissue using anti-SLC5A12 antibody. **(e)** Correlation plots of SLC5A12 expression with inflammatory mediator genes relevant to ELS formation and function. Gene expression was evaluated using real-time PCR (n = 18). Spearman correlation coefficient (r) and p-values (two-sided) are shown.

Here, we observed a progressive upregulation of chemokines and chemokine receptors responsible for T cell and B cell recruitment and organization (*Ccl19–Ccr7* and *Cxcl13–Cxcr5*) during ELS formation (from day 5 to day 12) (Fig. 1b and Extended Data Fig. 1a). Additionally, genes associated with the formation of functional ectopic germinal centres were found to be linked to the modulation of lactate metabolism in the SGs, including *Ldha* and *Ldhb*, as well as lactate transporters, as shown in Fig. 1b,c. In the context of germinal centre activity within ELS, T_FH_ cells, defined as CD4^+^, CXCR5^+^ and PD1^+^ T cells that support germinal centre B cell activation, are the primary source of IL-21 in the mouse SG. T_FH_ cells account for 30% of IL-21-producing CD4^+^ T cells, and the entire T_FH_ compartment produces IL-21 (Extended Data Fig. 1b).

To further explore the link between lactate metabolism and SG inflammation, we quantified lactate levels in both non-cannulated (control) and cannulated (experimental) mouse SG tissue. A significant increase in lactate concentration was observed in the cannulated group compared to non-cannulated controls (Fig. 1d). These findings reinforce the association between inflammatory processes and altered metabolic states in the SG, as evidenced by the upregulation of lactate transporters and metabolic enzymes linked to lactate in RNA-seq data.

To investigate whether the metabolic changes observed in the viral-induced model of SG ELS could be reproduced in SjD SG, we investigated the associations between the inflammatory response, lactate transporters and metabolic pathways in our bulk SG RNA-seq data from SjD SGs stratified for the presence of ELS (Fig. 1e). The unsupervised clustering of the SG transcriptomic profiles from patients with SjD versus sicca controls (patients with non-autoimmune sialadenitis) revealed a segregation of samples with higher expression of inflammatory mediators known to have a role in organizing inflammatory aggregates into ELS (Extended Data Fig. 1c). ELS-positive samples also showed higher expression of lactate transporters (Fig. 1f and Extended Data Fig. 1c). Inflammatory mediators, such as B cell–T cell chemokine-chemokine receptor axes (CCR7–CCL19, CXCR5–CXCL13) and cytokines (*IFNG*, *IL21*, *IL27*), were progressively upregulated in SjD gland tissue with increasing ELS organization from sparse immune cells (G0) to fully organized ELS (G3) (Fig. 1f and Extended Data Fig. 1c).

Examining lactate transporters from the SLC16 and SLC5A families, alongside metabolic enzymes upstream or downstream of lactate metabolism, we found a clear association between higher lactate transporter expression and ELS organization in SG tissue (Fig. 1f). This association was more pronounced in SjD SGs with ELS compared to SjD SGs without ELS or sicca SGs. Additionally, this was accompanied by the downregulation of metabolic enzymes (*ACACA*, *ACACB*, *PKM* and *FASN*), suggesting reduced usage of fatty acid or cholesterol synthesis from acetyl-CoA derived from lactate, and an increase in inflammatory cytokines (*IFNG* and *IL21*) and mediators (*STAT1* and *STAT3*) in SjD SGs with ELS, compared to SGs lacking this inflammatory feature or sicca controls (Fig. 1f and Extended Data Fig. 1c).

Of note, single-cell RNA-seq data from human SjD and sicca SGs revealed that lactate metabolism is broadly regulated across multiple cell types within the SGs (Fig. 1g). Specifically, we observed widespread expression of lactate dehydrogenase mRNA transcripts, with relatively lower expression in B cells and plasma cells. Notably, higher lactate dehydrogenase expression was detected in CD8^+^ T cells, fibroblast subtypes and myeloid cells in SjD compared to sicca.

### SLC5A12 expression in SjD correlates with key ELS drivers

Given that SLC5A12 expression was relatively low in bulk RNA-seq compared to other lactate transporters (a pattern often observed in publicly available RNA-seq datasets owing to its highly restricted and inducible expression), we further investigated lactate levels as well as SLC5A12 and cytokine expression in SGs from patients with SjD.

**Fig. 2:**
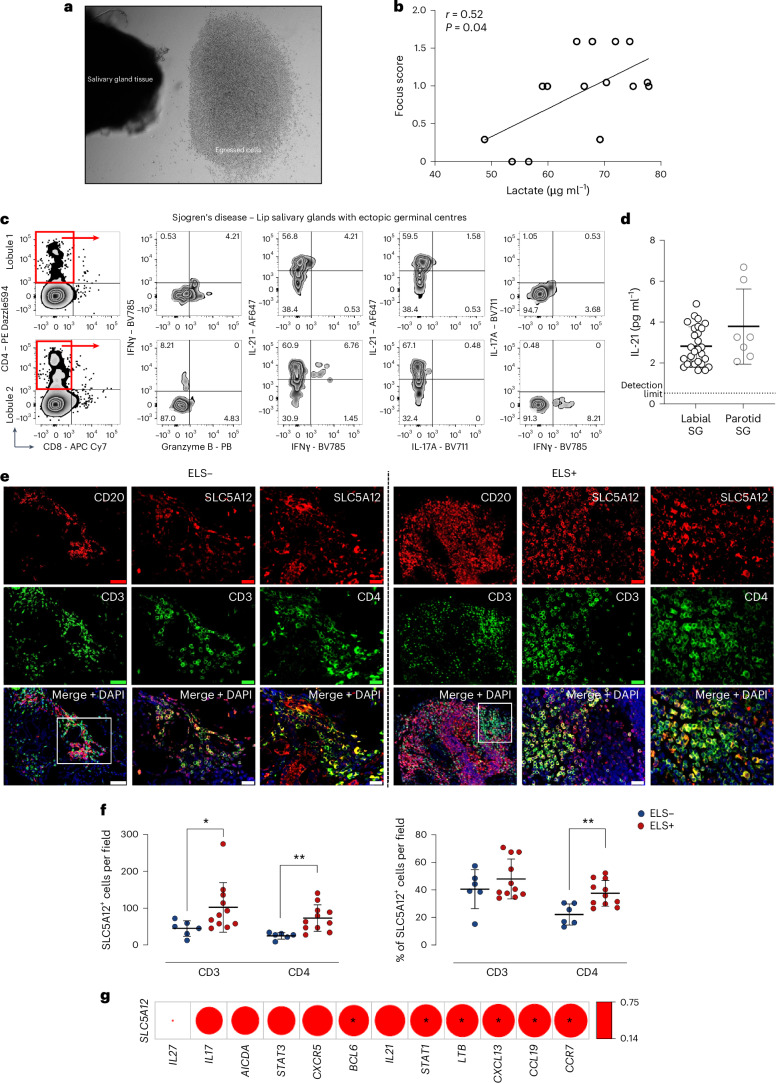
In vitro SG organ culture and immune cell dynamics in SjD. **a**, Representative images of the in vitro SG organ culture system showing a lip SG lobule from a patient with SjD and the immune cells spontaneously egressing from the tissue. **b**, Correlation plot of the lactate levels measured in the supernatant of the SG organ culture from an SjD patient and the focus score (a histopathological index of SG inflammatory infiltrate severity) of the corresponding lip SG lobule. Spearman correlation coefficient (*r*) and *P* value (two-sided) are shown. **c**, Representative flow cytometry plots showing cytokine production by CD4 T cells egressed from the lip SG in the organ culture system. Cytokine production is shown for T cells obtained from a lip SG with low inflammation (lobule 1, focus score of <1) and severe inflammation (lobule 2, focus score of >1.5). **d**, Detection of IL-21 in the supernatant of SG organ cultures derived from lip (*n* = 30) and parotid (*n* = 7) SGs of patients with SjD. **e**, Representative images of lip SGs from patients with SjD, classified based on the absence (ELS−) or presence (ELS+) of ectopic lymphoid structures. The left columns show B cell (CD20) and T cell (CD3) immune infiltration and their segregation. The middle and right columns depict SLC5A12 expression in all T cells (CD3) and specifically in T_H_ cells (CD4). Scale bars, 100 µm. **f**, Comparison of cell count (left) and percentages (right) of CD3 T cells and CD4 T_H_ cells expressing SLC5A12 in ELS− (*n* = 6) and ELS+ (*n* = 11) SGs from patients with SjD. Each dot represents an aggregate. Data are expressed as means, error bars, s.d. Statistical significance was determined using the Mann–Whitney *U*-test between groups (*P < 0.05, **P < 0.01). **g**, Correlation matrix of SLC5A12 expression with inflammatory mediator genes relevant for ELS formation and function. Gene expression was evaluated using real-time PCR (*n* = 18). The size of the circles represents the magnitude of each correlation, and the colour indicates the Spearman correlation coefficient (*r*). Statistically significant adjusted *P* values (adjusted for multiple testing with false discovery rate correction) are shown within the dots (*P < 0.05).

We set up SjD SG organ cultures (Fig. 2a) and measured lactate levels in the culture media, finding a correlation with the focus score (a histopathological index of SG inflammatory infiltrate severity that is currently part of the SjD classification criteria^47^ and strongly associated with lymphoma progression^48^) of the corresponding lip SG lobule (Fig. 2b). This analysis revealed increased lactate concentrations associated with a higher focus score. Furthermore, egressed cells were analysed by flow cytometry for key T cell cytokine expression (Fig. 2c), and IL-21 levels were quantified in the culture media using ELISA (Fig. 2d).

SjD SG tissue sections were screened for the presence of ELS and classified as ELS-positive or ELS-negative, based on the presence or absence of B cell (CD20) and T cell (CD3) segregation in discrete areas (Fig. 2e). This classification allowed us to specifically target tissues with and without organized immune cell aggregates for comparative analysis. Image analysis of the immunofluorescence staining revealed a significant upregulation of SLC5A12 in CD3 and CD4 T cells within ELS-positive SGs (Fig. 2e,f). To confirm the specificity of the antibody, we performed staining on kidney tissue, a well-established site of high SLC5A12 expression (Extended Data Fig. 1d). The antibody specifically labelled the proximal tubules, where SLC5A12 is predominantly localized, while showing no staining in the distal tubules. This selective staining pattern validates the antibody’s specificity in recognizing SLC5A12 within its expected anatomical context.

To ensure that the observed increase in SLC5A12 expression was not merely caused by a higher density of T cells in ELS-positive compared to ELS-negative tissues, we analysed the percentage of CD3 T cells and CD4 T_H_ cells expressing SLC5A12. The results showed that a significantly higher percentage of CD4 T_H_ cells expressed SLC5A12 in ELS-positive SGs compared to ELS-negative SGs (Fig. 2f). This finding underscores the preferential upregulation of SLC5A12 in the context of ELS formation.

Moreover, we performed quantitative PCR with reverse transcription to quantify SLC5A12 expression in SG tissues from patients with SjD and sicca controls. Our data demonstrated a positive correlation between SLC5A12 expression and the expression of several genes known to be involved in ELS organization and function (Fig. 2g and Extended Data Fig. 1e). These include CCR7–CCL19 and CXCL13, which are critical for B cell chemoattraction, as well as LTβ, which is crucial for lymphoid tissue organogenesis. Furthermore, we observed a positive correlation with genes involved in germinal centre activity, such as the transcription factor Bcl6 (Fig. 2g and Extended Data Fig. 1e), occurring with ELS. These correlations suggest that SLC5A12 might be involved in the metabolic reprogramming of T cells within ELS, potentially contributing to their maintenance and function.

### Lactate alters T_H_ cell profile, boosting IL-21 production

**Fig. 3:**
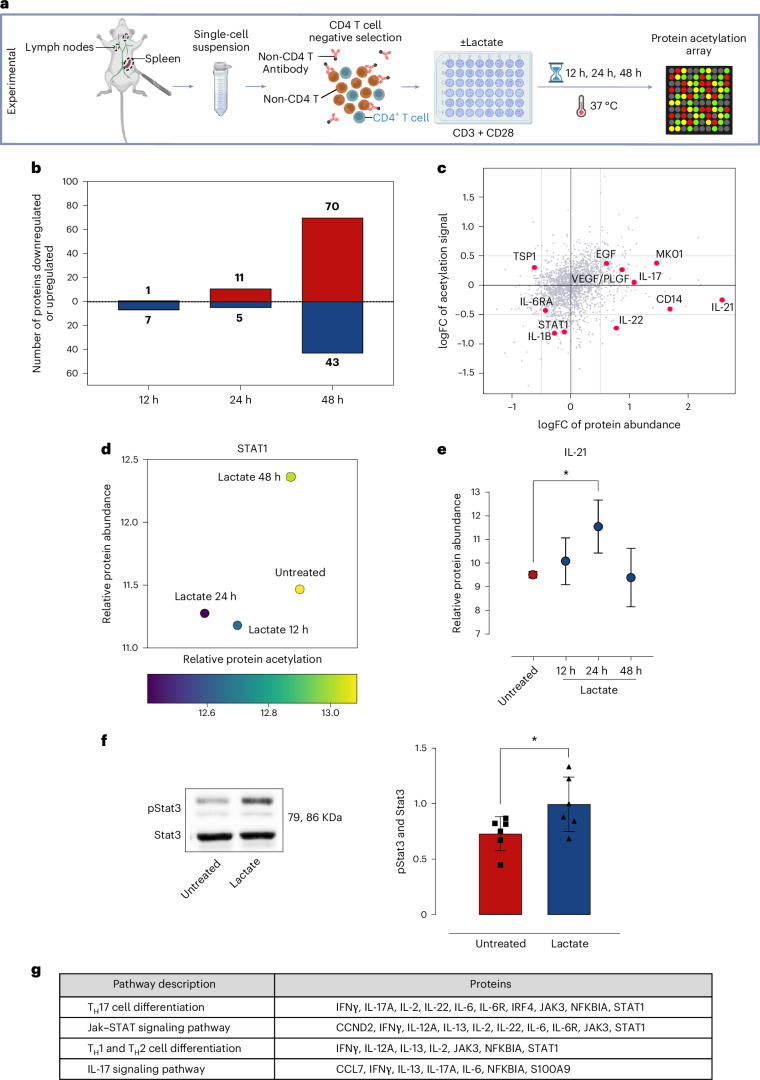
Acetylation study of murine CD4 T cells. **a**, Experimental workflow for the acetylation study of murine CD4 T cells. Lymph nodes and spleen were collected from mice and processed to obtain a single-cell suspension, from which CD4 T cells were isolated. The isolated CD4 T cells were then activated with CD3 and CD28 and treated with lactate or left untreated for 12, 24 and 48 h. The cells were then used to perform a protein acetylation array to assess abundance and levels of acetylation. **b**, Bar graph listing the number of antibodies with differential intensities when comparing untreated versus lactate-treated (12 h, 24 h and 48 h) CD4 T cells. For each comparison, red indicates upregulated proteins, while blue indicates downregulated proteins. **c**, Overview of differences in protein abundance and acetylation levels between untreated and lactate-treated (24 h) CD4 T cells. FC, fold change. **d**, Overview of differences in STAT1 abundance and acetylation levels between untreated and lactate-treated (12 h, 24 h and 48 h) CD4 T cells. **e**, Relative IL-21 levels in untreated and lactate-treated (12 h, 24 h and 48 h) CD4 T cells. For analysis of the samples, a one-factorial linear model was fitted with LIMMA, resulting in a two-sided *t*-test (*P < 0.05). Each sample was measured by four replicate spots per array (*n* = 3 biological replicates). **f**, Western blot analysis showing the levels of pStat3 and total Stat3 in CD4 T cells. The cells were either left untreated or treated with lactate for 24 h. Data are presented as mean values; error bars, s.d. Statistical significance was assessed using the Mann–Whitney *U*-test (*P < 0.05, *n* = 6 biological replicates). **g**, Selected KEGG pathways related to proteins with differential abundance and acetylation in untreated versus lactate-treated (24 h) CD4 T cells.

We next assessed whether lactate accumulation influences the autoimmune response, particularly T cell activation and differentiation. To understand how T cells respond in a lactate-rich environment, we activated murine CD4 T cells in the presence of lactate (10 mM) for 12, 24 or 48 h and compared them with CD4 T cells cultured in the absence of lactate. Protein extracts from these cells were subjected to an antibody-based protein array to assess the abundance and acetylation levels of 1,352 proteins (Fig. 3a).

**Extended Data Fig. 2:**
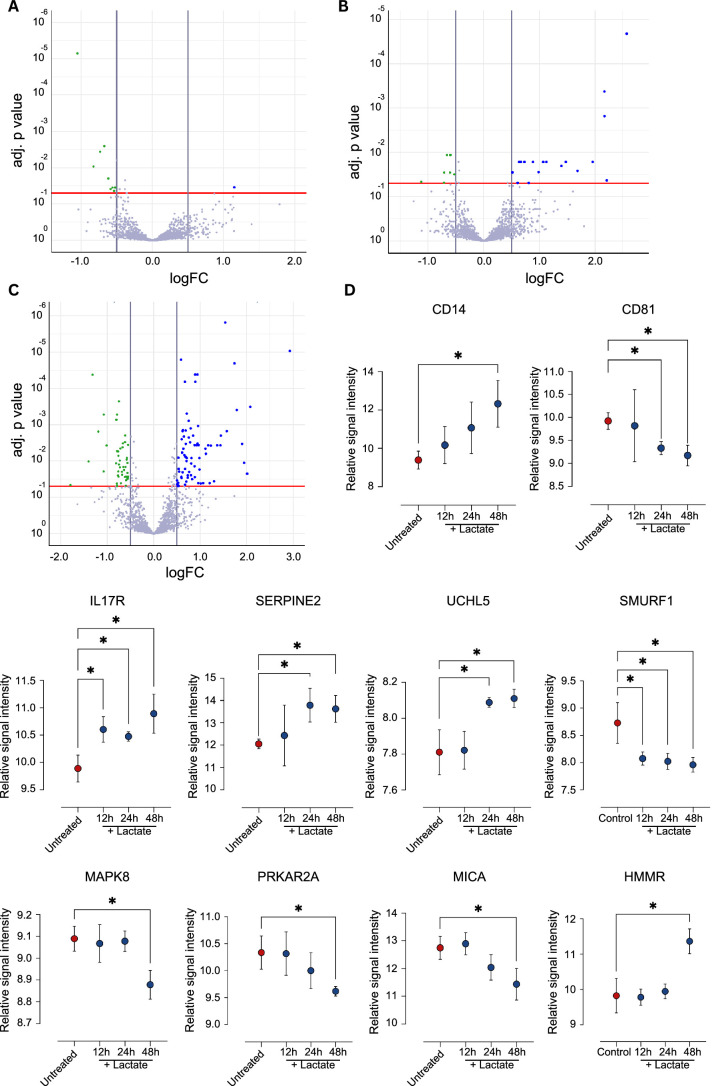
Protein expression analysis. Volcano plots illustrating the results of the differential expression analysis comparing untreated and lactate-treated (12 h **a**, 24 h **b**, and 48 h **c**) CD4 T cells. Each point on the plot represents a single protein. P values (adjusted for multiple testing) and corresponding log-fold changes (logFC) are shown. A significance level of adj. p-value = 0.05 is indicated as a horizontal red line. The logFC cutoffs are indicated as vertical lines. Proteins with a positive logFC had a higher abundance in lactate-treated samples, proteins with a negative value in untreated samples. Proteins indicated with green points feature a logFC > 1, while not reaching the significance threshold. **(d)** Individual array values for a set of differential proteins in untreated and lactate-treated (12 h, 24 h, and 48 h) CD4 T cells. Each sample is measured by four replicate spots per array. For analysis of the samples a one-factorial linear model was fitted with LIMMA resulting in a two-sided t-test. Data are presented as mean values +/- SD (*p < 0.05, n = 3 biological replicates).

**Extended Data Fig. 3:**
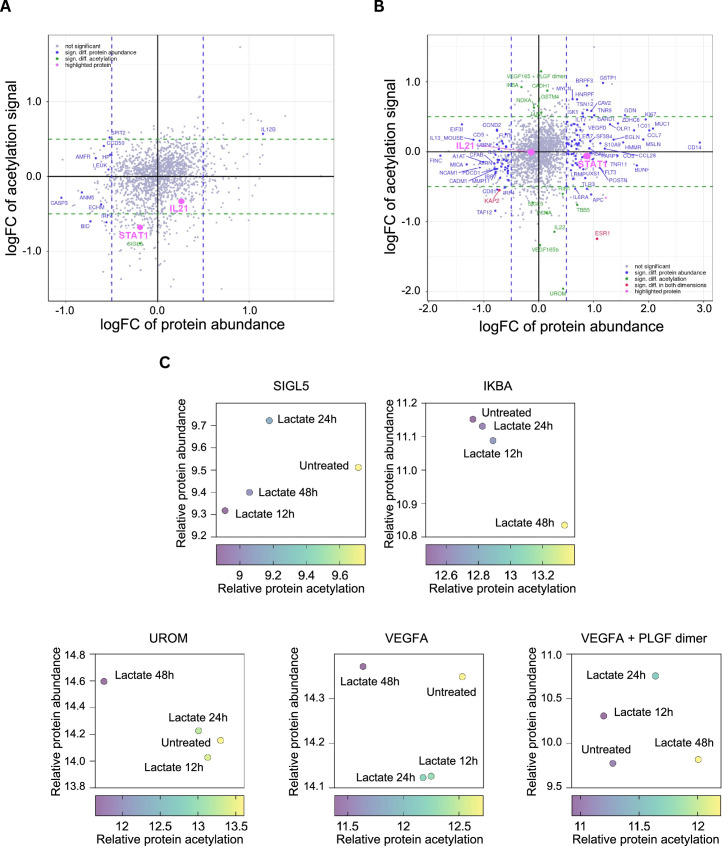
Differential protein abundance versus acetylation level. Overview of differences in protein abundance as well as acetylation levels between untreated and lactate-treated (12 h **a**, and 48 h **b**) CD4 T cells. Proteins with a positive logFC (protein, x-axis) were more abundant in lactate-treated samples, while proteins with a negative logFC were more abundant in untreated samples. For proteins with a positive logFC (acetyl, y-axis), higher acetylation signals were obtained in lactate-treated samples. For proteins with a negative logFC, higher acetylation signals were obtained in untreated samples. **(c)** Individual array values for a set of differential proteins in untreated and lactate-treated (48 h) CD4 T cells.

Here, our analysis revealed that approximately 10% of proteins had altered expression after 48 h of lactate stimulation, with 70 proteins upregulated and 43 downregulated (Fig. 3b, Extended Data Fig. 2a–d and Supplementary Data Tables 1–3), suggesting that lactate acts as a master regulator of protein expression in activated CD4 T cells under in vitro conditions, mimicking inflamed tissue. Notably, several inflammatory factors were affected, with IL-17, IL-21 and IL-22 levels being upregulated, and alterations observed in acetylation levels for IL-6RA, STAT1 and IL-1B (reduced) and TSP1 (increased) (Fig. 3c, Extended Data Fig. 3a–c and Supplementary Data Tables 4 and 5).

Additionally, STAT1 acetylation was reduced at 12 and 24 h of lactate treatment but increased again at the 48 h time point, concomitantly with increased abundance (Fig. 3d). Correspondingly, IL-21 abundance increased during periods of STAT1 deacetylation and decreased once STAT1 acetylation was restored (Fig. 3e). IL-21 is known to regulate STAT3, and consistent with IL-21 upregulation at 24 h, we observed increased phosphorylation of STAT3 (Fig. 3f).

KEGG pathway analysis identified T_H_17 (IL-17/IL-22) and T_H_1 and T_H_2 pathways as the main pathways activated by lactate treatment in CD4 T cells activated in vitro (Fig. 3g). This suggests that lactate not only influences protein expression and acetylation but also modulates key inflammatory pathways in T_H_ cells, promoting a T_H_17/T_H_1 response and highlighting its potential role in autoimmune responses.

### B cell depletion in SjD alters SG ELS with lactate and enzyme modulation

We recently demonstrated that immunomodulating agents, such as the B cell depleting agent (monoclonal anti-CD20 antibody, rituximab), effectively prevented or reduced ELS formation within SjD SGs compared to the placebo-treated group^49^.

**Fig. 4:**
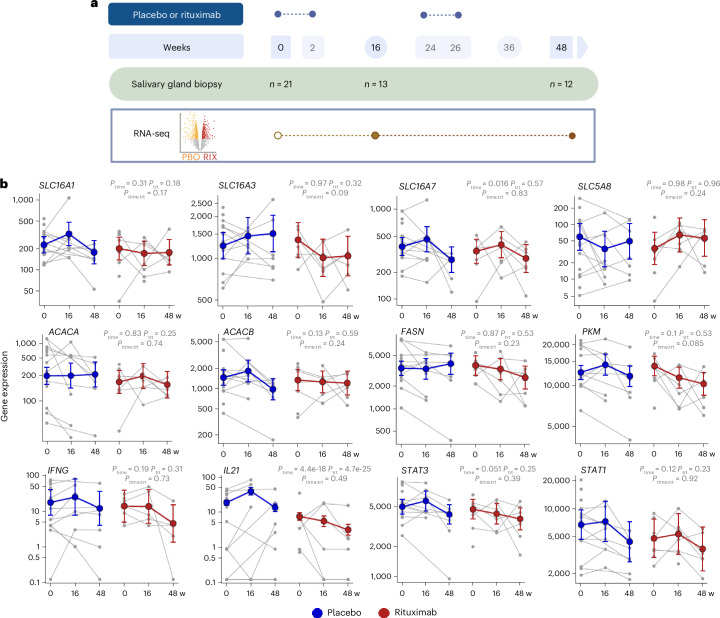
Longitudinal transcriptomic profiling of SG tissue in the TRACTISS trial. **a**, Schematic of the longitudinal transcriptomic study from the TRACTISS randomized clinical trial in patients with SjD, including sequential lip biopsies from patients randomized to either placebo (PBO) or rituximab (RIX) (anti-CD20 B cell depleting agent) treatment. **b**, Changes over time in the expression of genes for lactate transporters, metabolic enzymes downstream of lactate signalling and inflammatory mediators. Blue (placebo) and red (rituximab) lines represent the fitted negative binomial mixed-effects model with a 95% confidence interval. The *P* values of the week/treatment ratio interaction model are shown on top. Data from patients with SjD treated with placebo (*n* = 12) or rituximab (*n* = 8) are included.

Hence, we next investigated whether modulation of inflammation in SjD SGs through rituximab was linked to changes in lactate transporters and metabolic enzymes. To address this question, we analysed data from the TRACTISS randomized clinical trial in SjD, which provides comprehensive longitudinal transcriptomic data on SG tissue (Fig. 4a).

Importantly, decreased recruitment of B cells, diminished T_FH_ cell differentiation and lower levels of IL-21 were accompanied by a progressive downregulation of lactate transporters and related metabolic enzymes over time (Fig. 4b), indicating a shift in the metabolic needs of the SG tissue in response to immunomodulatory therapy affecting ELS organization and function.

### Lactate sustains IL-21 in SjD, reversed by SLC5A12 mAb blockade

**Fig. 5:**
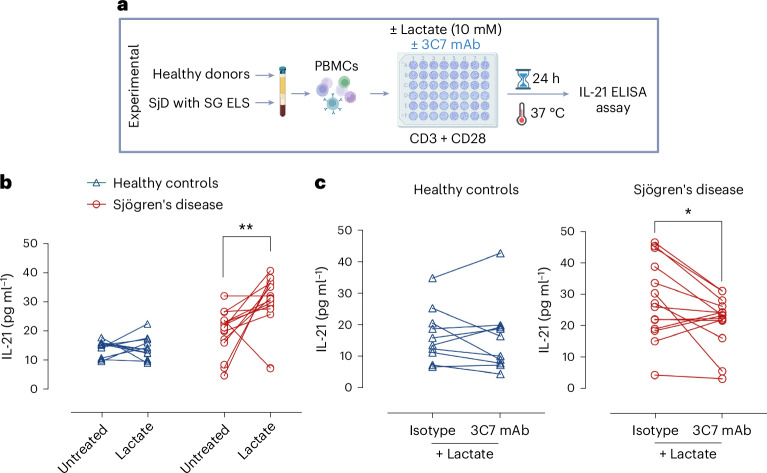
Quantification of IL-21 in ex vivo PBMCs from patients with SjD. **a**, Schematic representation of the IL-21 quantification workflow from ex vivo supernatant. **b**, Levels of IL-21 in culture supernatant of PBMCs from patients with SjD with SG ELS (*n* = 15) and from healthy donors (*n* = 12), stimulated in the absence and presence of lactate (10 mM). Lines connect dots representing PBMC samples treated with lactate or left untreated from the same donor. Statistical analysis was performed using the Wilcoxon signed-rank test (*P < 0.05, **P < 0.01). **c**, Levels of IL-21 in culture supernatant of PBMCs from patients with SjD, stimulated with lactate (10 mM) and treated with either anti-SLC5A12 3C7 mAb or its isotype control. Lines connect dots representing PBMC samples treated with anti-SLC5A12 mAb and its isotype control from the same donor. Statistical analysis was performed using the Wilcoxon signed-rank test; *P* value (two-sided) is shown (*P < 0.05).

Given the close association between IL-21, lactate transporters and metabolic changes that we observed in SjD SG with ELS, we next evaluated the effect of lactate on IL-21 expression in peripheral blood mononuclear cells (PBMCs) isolated from patients with SjD who had biopsies positive for ELS (Fig. 5a). These patients were selected because of their high circulating levels of IL-21, which we recently showed to be associated with a higher frequency of circulating T_FH_ cells displaying aberrant IL-21 secretion^20^.

Lactate stimulation led to a significant increase in IL-21 secretion in PBMCs from patients with SjD, surpassing the high levels they expressed basally, while no such effect was observed in cells from healthy controls (Fig. 5b). Furthermore, blocking SLC5A12 with the specific monoclonal antibody (mAb) 3C7 reduced IL-21 production compared to the isotype control, and this effect was again noted exclusively in PBMCs from patients with SjD (Fig. 5c), highlighting the potential specificity of SLC5A12 blockade.

Overall, these data suggest that lactate promotes or sustains elevated IL-21 production by CD4 T cells, which are primarily T_FH_ cells in SjD. The findings indicate that lactate might contribute to the pathophysiology of SjD by enhancing the inflammatory response mediated by T follicular cells.

### SLC5A12 blockade impacts ELS in an inducible Sjögren’s model

**Fig. 6:**
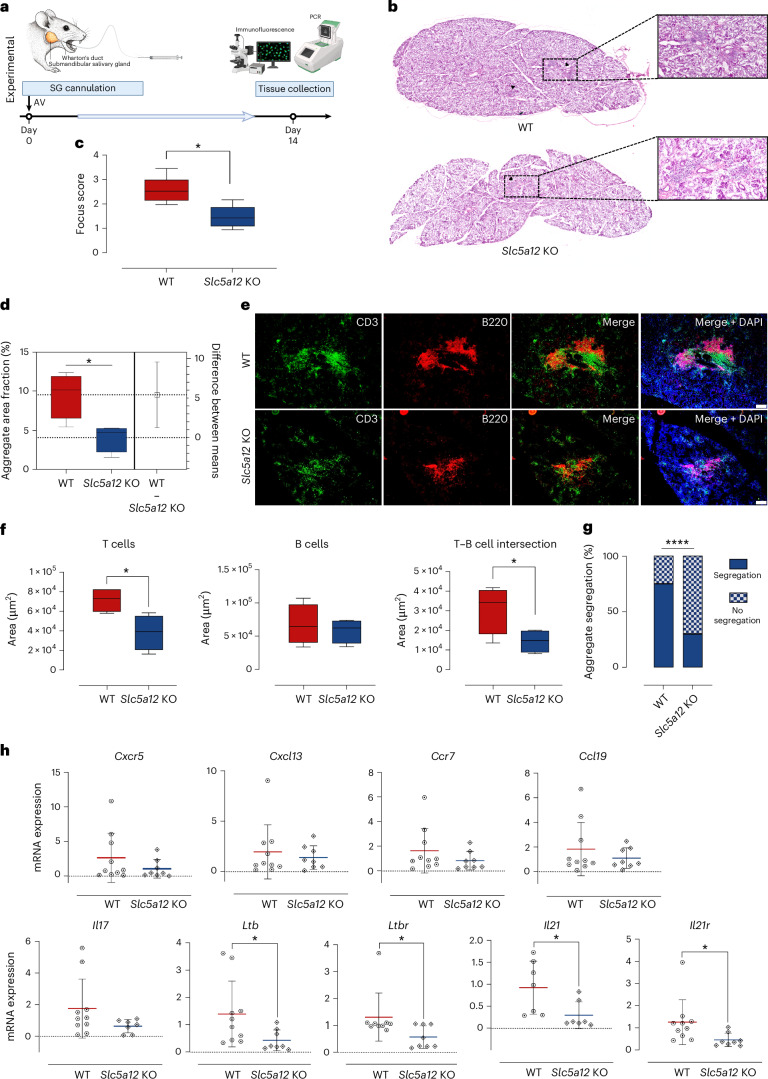
Histological analysis, immune characterization and gene expression quantification in wild-type and *Slc5a12* KO murine SGs. **a**, Workflow of the cannulation of murine SGs in wild-type (WT) and *Slc5a12* knockout (KO) mice model showing a representation of the mouse SG, and the cannula used for precise delivery of the AV into the gland. The experimental timeline shows that the AV was injected into the SG on day 0, and on day 14, SG tissues were collected for subsequent analyses. Post collection, tissues were processed for immunofluorescence and qPCR to assess relevant biomarkers and gene expression levels. **b**, Representative images of hematoxylin and eosin (H&E) staining of the whole SGs from WT and *Slc5a12* KO mice. Black arrows point to inflammatory foci (peri-ductal leukocytic infiltrates with more than 50 lymphocytes). **c**, Comparison between WT and *Slc5a12* KO mice (*n* = 5 per group) of focus score calculated on H&E images. **d**, Aggregate area fraction (% of SG area occupied by the inflammatory infiltrate), measured on immunofluorescence stainings. **e**, Representative immunofluorescence staining of SG tissue sections from WT and *Slc5a12* KO mice. CD3 (green) is used as a marker for T cells, B220 (red) is used as a marker for B cells and DAPI (blue) is used as a marker for nuclei. The images reveal the distribution and localization of T cell and B cell inflammatory aggregates in the tissue. Scale bars, 100 µm. **f**, Comparison between WT and *Slc5a12* KO mice of positive area for CD3 (T cells), B220 (B cells) and both (T cell–B cell intersection), respectively, as calculated from **e**. Data are presented as means; error bars, s.d. (*n* = 4 biological replicates per group). Statistical analysis was performed using an unpaired *t*-test. In **c**, **d** and **f**, box and whisker plots show the 75^th^ and 25^th^ percentiles of the data, and minimum and maximum values. Statistical significance was determined using a Mann–Whitney *U*-test (two-sided). **g**, Prevalence of segregated and non-segregated aggregates over the total number in WT and *Slc5a12* KO mice, as calculated from **e**. **h**, qPCR analysis of gene expression levels for *Cxcr5, Cxcl13, Ccr7, Ccl19, Ltb, Ltbr, Il17, Il21* and *Il21r* in WT and *Slc5a12* KO mice. Gene expression levels are normalized to a housekeeping gene and presented as relative expression levels. Data are expressed as means from *n* = 8–10 mice per group; error bars, s.d. Statistical significance was determined using a Mann–Whitney *U*-test (two-sided) and outliers were excluded by Grubb’s test (*P < 0.05, **P < 0.01, ***P < 0.001, ****P < 0.0001).

**Fig. 7:**
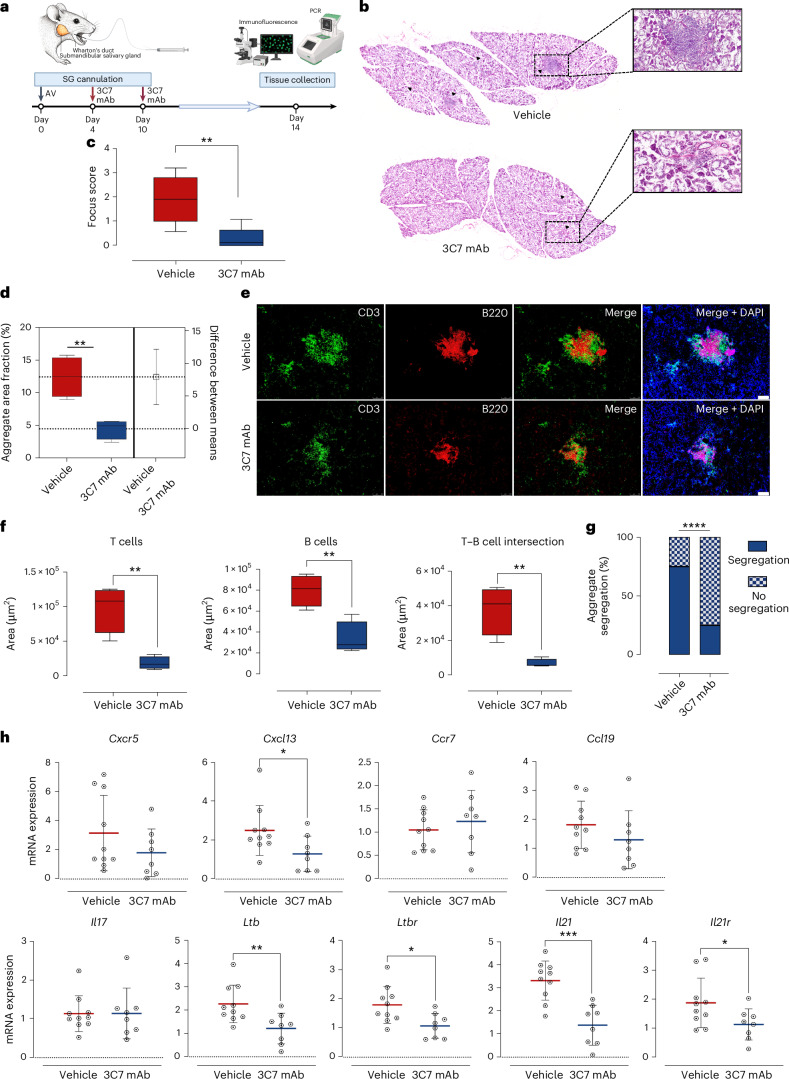
Histological analysis, immune characterization and gene expression quantification in vehicle and 3C7 mAb-injected murine SGs. **a** Workflow of the mouse inducible ELS formation, following SGs cannulation. The experimental timeline shows that the AV was injected into the SG on day 0; on days 4 and 10, the 3C7 monoclonal anti-Slc5a12 antibody was administered. On day 14, SG tissues were collected for subsequent analyses. Post collection, tissues were processed for immunofluorescence and qPCR to assess relevant biomarkers and gene expression levels. **b**, Representative images of H&E staining of the whole SGs from vehicle-cannulated and 3C7 mAb-cannulated mice. Black arrows point to inflammatory foci (peri-ductal leukocytic infiltrates with more than 50 lymphocytes). **c**,**d**, Comparison between vehicle-cannulated and 3C7 mAb-cannulated mice (*n* = 5 or 6 per group) of focus score calculated on H&E images (**c**) and aggregate area fraction (% of SG area occupied by the inflammatory infiltrate), calculated on immunofluorescence images (**d**). **e**, Representative immunofluorescence staining of SG tissue sections from vehicle and 3C7 mAb-injected mice. CD3 (green) is used as a marker for T cells, B220 (red) is used as a marker for B cells and DAPI (blue) is used as a marker for nuclei. The images reveal the distribution and localization of T cells and B cells in the tissue and their segregation. Scale bars, 100 µm. **f**, Comparison between vehicle and 3C7 mAb-cannulated mice of the positive area for CD3 (T cells), B220 (B cells) and both (T cell–B cell intersection), respectively, as calculated from **e**. Data are presented as means; error bars, s.d. (*n* = 4 biological replicates per group). Statistical analysis was performed using an unpaired *t*-test. In **c**, **d** and **f**, box and whisker plots show the 75^th^ and 25^th^ percentiles of the data, and minimum and maximum values. Statistical significance was determined using a Mann–Whitney *U*-test (two-sided). **g**, Prevalence of segregated and non-segregated aggregates over the total number in vehicle and 3C7 mAb-cannulated mice, as calculated from **e**. **h**, qPCR analysis of gene expression levels for *Cxcr5, Cxcl13, Ccr7, Ccl19, Ltb, Ltbr, Il17, Il21* and *Il21r* in vehicle and 3C7 mAb-injected mice. Gene expression levels are normalized to a housekeeping gene and presented as relative expression levels. Data are expressed as means from *n* = 8–10 mice per group; error bars, s.d. Statistical significance was determined using a Mann–Whitney *U*-test and outliers were excluded by Grubb’s test (*P < 0.05, **P < 0.01, ***P < 0.001, ****P < 0.0001).

**Extended Data Fig. 4:**
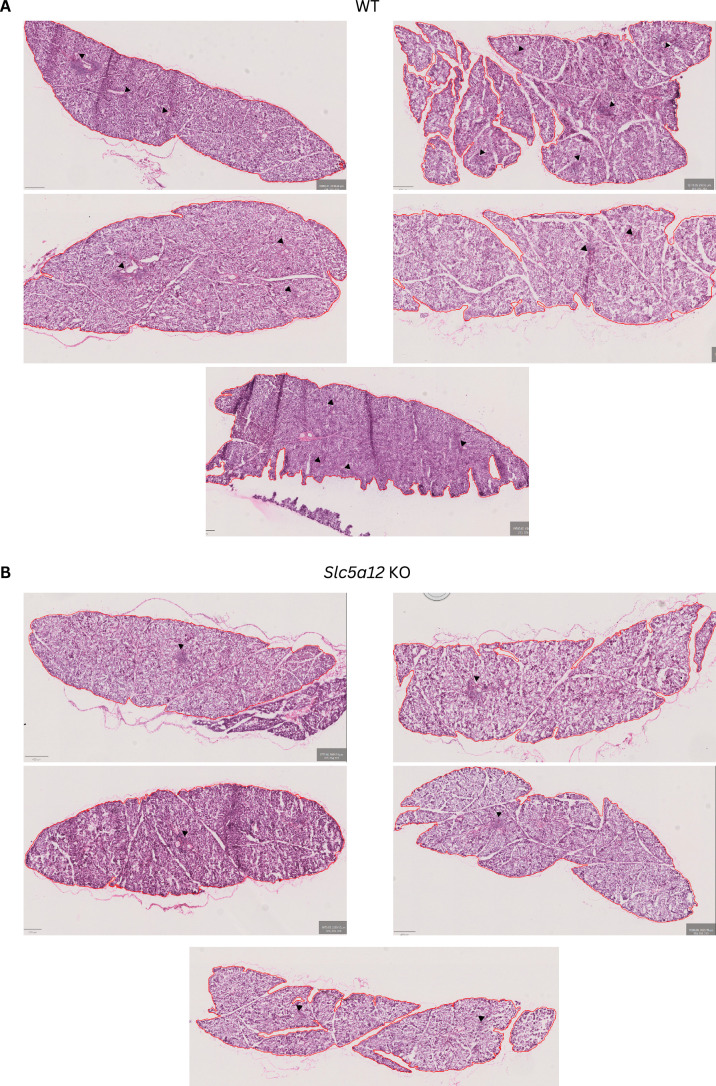
Comparison of inflammatory foci in salivary glands of WT and *Slc5a12* KO mice. Whole-slide images of hematoxylin and eosin (H&E) staining of salivary glands from WT **(a)** and Slc5a12 KO **(b)** mice (n = 5 per group), showing differences in the inflammatory foci (peri-ductal leukocytic infiltrates containing more than 50 lymphocytes, black arrows).

We next evaluated the formation and function of inflammatory infiltrates and ELS through the genetic or pharmacological blockade of SLC5A12 in the viral-induced murine model of sialadenitis, which mimics focal lymphocytic aggregation and ELS in SjD (Figs. 6a and 7a). Deletion of *Slc5a12* in mice led to significant reduction in inflammatory aggregates (Fig. 6b and Extended Data Fig. 4a,b) as demonstrated by a reduced SG focus score (Fig. 6c) and aggregate area fraction (Fig. 6d), both of which are used as histopathological indexes of SG inflammatory infiltrate severity in clinical practice^50^. We also observed clear alterations in ELS formation (Fig. 6e), characterized by a reduced T cell area, a trend toward a reduced B cell area and decreased B cell–T cell intersection, indicating the extent of B cell–T cell interactions (Fig. 6f), as assessed by an automated image analysis software we developed. Furthermore, we observed a decreased B cell–T cell segregation (Fig. 6g). RT–qPCR analysis of SG tissues showed impacts on *Ltb* and *Ltbr*, as well as *Il21* and *Il21r*, with trends indicating a reduction in various inflammatory genes associated with ELS, including chemokines and chemokine receptors (Fig. 6h). Interestingly, in this model, which is not IL-17-dependent owing to the strong restriction of IL-17 expression by myeloid-cell derived IL-27 (ref. ^12^), the deletion of *Slc5a12* did not significantly affect *Il17* expression (Fig. 6h).

**Extended Data Fig. 5:**
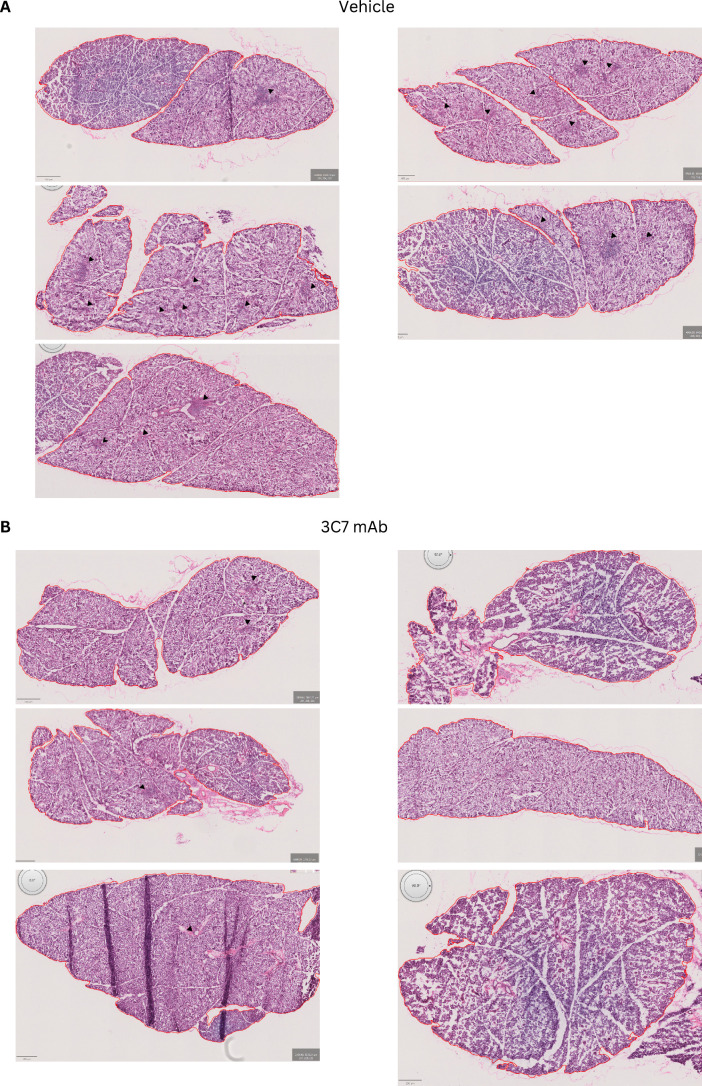
Comparison of inflammatory foci in salivary glands of vehicle-cannulated and 3C7 mAb-cannulated mice. Whole-slide images of hematoxylin and eosin (H&E) staining of salivary glands from vehicle-cannulated **(a)** and 3C7 mAb-cannulated **(b)** mice (n = 5/6 pr group), showing differences in inflammatory foci (peri-ductal leukocytic infiltrates containing more than 50 lymphocytes, black arrows).

Similarly, pharmacological blockade of SLC5A12 with the 3C7 mAb, administered therapeutically in two doses on days 4 and 10 of the inducible SjD model (Fig. 7a), had a profound impact on inflammatory aggregates (Fig. 7b and Extended Data Fig. 5a,b) as demonstrated by a reduced SG focus score (Fig. 7c) and aggregate area fraction (Fig. 7d). This treatment led to alterations in ELS formation (Fig. 7e), characterized by decreased areas occupied by both T cells and B cells, reduced B cell–T cell intersection area (Fig. 7f) and decreased B cell–T cell segregation (Fig. 7g). Again, RT–qPCR of SG tissues from ELS areas showed impacts on *Ltb, Ltbr*, *Il21, Il21r* and *Cxcl13*, with trends towards a reduction in various inflammatory genes associated with ELS, including other chemokines and chemokine receptors (Fig. 7h). Consistent with the genetic model, blocking *Slc5a12* did not significantly impact *Il17* expression (Fig. 7h), reinforcing the selectivity of SLC5A12 targeting.

Overall, these data demonstrate that lactate accumulation in the local SG microenvironment functionally regulates ELS development and function through sensing by lactate transporters on immune cells, primarily infiltrating CD4 T cells. The functional impact of lactate and its transporters on T_H_ subsets is particularly evident for IL-21 modulation, a key cytokine involved in germinal centre formation and B cell differentiation. These findings highlight the critical role of lactate in sustaining the inflammatory environment in SjD that favours ELS development and suggest that targeting the lactate transporter SLC5A12 could be a promising therapeutic strategy for resolving ELS.

## Discussion

Lactate build-up has been observed in numerous disease microenvironments, ranging from inflamed tissues in autoimmune and cardiovascular diseases to tumour microenvironments, infected lungs and obese abdominal adipose tissue^37^. Chronic lactate accumulation at these sites is associated with disease progression; however, the mechanisms induced by lactate may be context-dependent. In cancer, lactate has been linked to the promotion of immune suppression, including the induction of regulatory T cells^34^ and anti-inflammatory macrophages, fostering an environment permissive to further tumour growth^51^. However, in autoimmune tissues, such as rheumatoid arthritis, lactate produced by synovial fibroblasts is taken up by infiltrating CD4 T cells^44^ or γδ T17 cells^45^. This uptake leads to increased production of IL-17 by T_H_ cells, which further promotes the inflammatory autoimmune process^44,45^. Similarly, SG epithelial cells from patients with SjD, targets of autoimmune reactions and active participants in sustaining inflammation, showed increased expression of glucose transporter-1 (GLUT-1) upon activation, suggesting a metabolic shift towards glycolysis, where lactate is the main end product^52^.

ELS have been identified in the microenvironments of various diseases, including autoimmune disorders, cancer, cardiovascular conditions and infectious diseases^53^. These aggregates of B cells and T cells possess specialized functions, including the production of autoantibodies, inflammatory cytokines and chemokines, which contribute to the disease process^1^. The main drivers of ELS formation are LTb and LTbr^54^. Additionally, chemokines, chemokine receptors and inflammatory cytokines have been implicated in ELS formation and maintenance^54^. Whether metabolic processes are involved in the organization or function of ELS is currently unknown. We and others have shown that lactate promotes the entrapment of immune cells at sites of inflammation and metabolic reprogramming, leading to increased IL-17 production^44^. Both signals are potentially important in the steps of ELS organization. Hence, we reasoned that lactate could serve as a master regulator of ELS formation in inflamed tissues.

Indeed, our study shows that lactate transporters are preferentially upregulated in the SG of SjD with ELS, associating with the modulation of metabolic and inflammatory genes, particularly *IL21* and *STAT1*. These genes are involved in the signals of ELS organization in SjD SGs^49^, as seen in human biopsies from the largest UK cohort of patients with SjD and the TRACTISS trial, critically linking lactate and lactate transporters to disease severity in SjD.

The lactate levels in autoimmune-inflamed tissues, such as the SG in SjD or the synovial joint in rheumatoid arthritis, typically range between 10 mM and 15 mM, compared to 1–2 mM in the blood of healthy individuals. This increase in lactate contributes to a lower pH in the inflamed tissue, although it generally does not fall below pH 6.9. Given that the pKa of lactate is 3.83, very little lactic acid will be present in the tissue, with sodium lactate being the predominant form. Therefore, lactate-H^+^ transporters like MCT1 and MCT4 are not likely to have a substantial role in this context, whereas a Na^+^-lactate transporter such as SLC5A12 is likely to be more relevant. By contrast, in the tumour microenvironment, lactate concentrations can reach up to 40 mM, resulting in a more pronounced pH drop (down to around pH 6), which may enhance the relevance of lactate-H^+^ symporters. Furthermore, although MCT1 and MCT4 are expressed in most tissues and are highly expressed under steady-state conditions, SLC5A12 expression is normally restricted to the proximal tubules of the kidney, small colon and retina^55^. In the context of inflammation, however, SLC5A12 is upregulated in specific immune cell subsets, such as CD4^+^ T_H_17, T_FH_ and B cells, and is only expressed within the diseased tissue. Previous studies have demonstrated that blockade of SLC5A12 can have functional and disease-improving effects^27,43,44^. Conversely, SLC5A12 expression in CD8^+^ T cells is minimal, and its blockade does not appear to affect macrophage function.

Given that this upregulation is tissue-specific and not observed in peripheral tissues^44^, we believe targeting SLC5A12 could yield therapeutic benefits while minimizing side effects, in contrast to the adverse outcomes observed in clinical trials targeting MCT1.

ELS have been reported in cancer, where they have been associated with clinical response to PD1 immune checkpoint blockade^47^. However, to the best of our knowledge, no current evidence links metabolic reprogramming to the formation of ELS in cancer, and it is possible that such drivers are specific to the tumour microenvironment.

Furthermore, we reveal that lactate acts as a master regulator of the expression of about 10% of the investigated proteome of activated CD4 T cells in conditions mimicking the build-up of lactate in the inflamed tissue and modifies the acetylation levels of several targets. One important pathway regulated by lactate is the STAT–IL-21 axis. Lactate induces IL-21 production in PBMCs from patients who are ELS-positive, and blocking SLC5A12 with a mAb (3C7) that we have generated and are developing for therapy results in reduced levels of IL-21.

Together with the reduction of IL-21 and induction of ELS resolution, we have previously shown egress of immune cells from the inflamed site and reduction of IL-17. Therefore, SLC5A12 blockade shows multidimensional beneficial effects which are reminiscent of blockade of PD1 and CTLA4 in immuno-oncology, leading us to propose that SLC5A12 represents an important example of a checkpoint in inflamed tissues in autoimmune diseases.

Being solely expressed at high levels in the diseased tissue^44^, SLC5A12 also represents an ideal target to avoid widespread immune suppression and unnecessary targeting of peripheral immune cells. We tested whether targeting SLC5A12 genetically or pharmacologically would modulate ELS aggregation in a viral-induced inducible murine model of ELS within the SGs mimicking SjD^6^. Strikingly, both approaches led to a significant reduction of ELS aggregation, with downregulation of both effects, particularly on LTb and IL-21 pathways, suggesting a role for lactate both in the formation and function of ELS. We previously showed that in this model, ELS formation is independent of IL-17 (ref. ^14^) because of restriction of IL-17 expression by IL-27 (ref. ^12^). Hence, the evidence that SLC5A12 blockade has no effect on healthy donors or on IL-17 outputs in the murine model of SjD is particularly important, as it suggests that targeting SLC5A12 results in beneficial immunomodulatory effects dependent on the local inflammatory microenvironment. Additionally, evidence that lactate and SLC5A12 blockade selectively modulate activated CD4 T_H_ cells in SjD but not in healthy donors demonstrates selectivity of action, which might reduce off-target detrimental effects, such as compromising T_H_17 immunity required for skin and gut homeostasis.

**Extended Data Fig. 6:**
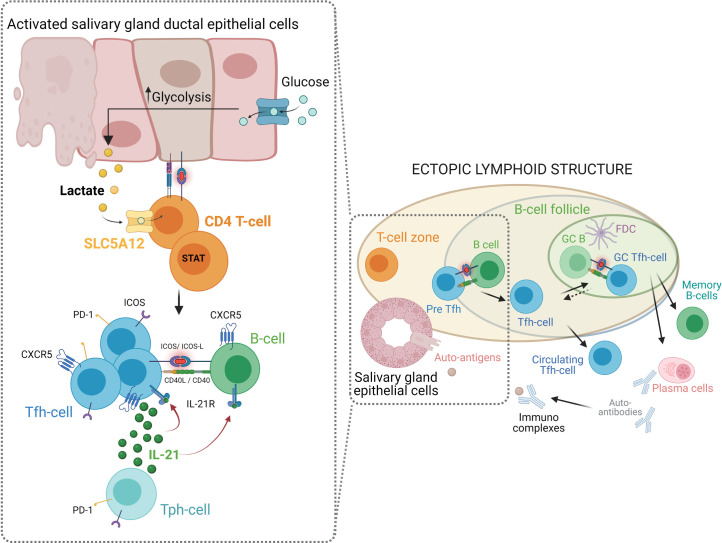
Mechanisms of ectopic lymphoid structure formation in salivary glands. The figure illustrates the proposed impact of lactate accumulation on T cell activation and the development of ectopic lymphoid structures in SjD SGs. (Left panel) Activated stromal cells, such as ductal epithelial cells, and infiltrating immune cells increase glycolysis, leading to lactate production and release into the microenvironment. CD4^+^ T cells uptake lactate via the SLC5A12 transporter. Lactate stimulates IL21 production by Tfh and Tph cells, driving B-cell activation and differentiation. (Right panel) Within the ectopic lymphoid structure, B cells organize into follicles supported by follicular dendritic cells (FDCs). Tfh cells provide help to B cells, leading to the production of autoantibodies and the generation of plasma cells and memory B cells. Autoantigens from the salivary gland epithelial cells contribute to the formation of immune complexes, perpetuating the autoimmune response.

Overall, our data demonstrate a previously unrecognized metabolic control of ELS organization, showing that the metabolite lactate acts as a master regulator of ELS formation through modulation of tissue-specific CD4 T cell functions (Extended Data Fig. 6). Additionally, our findings suggest that the inducible SLC5A12 lactate transporter represents an important checkpoint in autoimmunity, whereby both gene targeting and pharmacological blockade plastically modulate key inflammatory pathways that are specific to the local microenvironment in which ELS develop. Hence, therapeutic interventions with the capacity to interfere with lactate-driven immunopathology may impact not only autoimmune diseases such as SjD but also other conditions in which ELS exert a key role in orchestrating disease-specific local immune responses, including cancer, infection and graft rejection.

## Methods

### Human samples

The current study includes two distinct SjD cohorts: an observational disease–control cohort (SjD–sicca) and the TRACTISS randomized clinical trial, comparing rituximab versus placebo treatment in patients with SjD (Supplementary Data Table 6).

The human study, including the disease–control cohort (SjD–sicca), was approved by the Research Ethics Committee (reference 17/WS/0172), West of Scotland REC 4 and the Research Ethics Committee (reference 05/Q0702/1), NRES Committee London–Westminster. All patients provided written consent according to the principles of the Declaration of Helsinki. Labial SG biopsies were collected after informed consent from patients displaying xerostomia and xerophthalmia and meeting the 2016 American–European Consensus Group (AECG) criteria for SjD^47^, alongside patients with non-specific chronic sialadenitis (sicca, controls).

The TRACTISS randomized clinical trial was approved by the Leeds Research Ethics Committee (10/H1307/99). A cohort of patients with SjD (*n* = 133) received either two doses, 2 weeks apart, of intravenous rituximab (1,000 mg) or placebo (250 ml saline) in two courses at weeks 0, 2 and 24, 26, in combination with corticosteroid in both arms. A subset of the patients with SjD part of the TRACTISS study (*n* = 29) consented for a lip biopsy at weeks 0, 16 and 48, providing written informed consent^47^.

### Animal experiments

The study was approved by the institutional Animal Welfare and Ethical Review Body (UoB PPL PP1011240 and QMUL PPL P29EDC088, Home Office). Young adult male C57BL/6 mice (Charles River Laboratories) and *Slc5a12* knockout mice (produced at the Sanger Institute by CRISPR–Cas9), aged between 10 and 13 weeks at the start of the experiments, were housed in a specific-pathogen-free animal facility under standard conditions. Animals were maintained in ventilated cages with a 12 h light–dark cycle, at a constant temperature of 22 ± 2 °C and relative humidity of 50 ± 10%. Mice had ad libitum access to food and water. Environmental enrichments were added to all cages. Genotyping of ear notches taken at weaning was performed by Transnetyx. Mice were randomly assigned to experimental groups, ensuring that the age and weight were matched between groups. Mice were anaesthetized with ketamine (60 mg kg^−1^) and xylazine (12 mg kg^−1^) administered intraperitoneally. Analgesia was provided as 0.1 mg kg^−1^ buprenorphine hydrochloride by subcutaneous injection. The submandibular gland was cannulated through the excretory duct using heat-drawn glass gas chromatography tubing (0.1 mm internal diameter; Sigma-Aldrich). Approximately 20 µl of AdV solution, equivalent to 1 × 10^8^ plaque-forming units of reporter-encoding AdV or buffer vehicle, was injected into the submandibular gland using an attached Hamilton syringe (Sigma-Aldrich). The same volume of the vehicle buffer was delivered as a negative control. In the treated group, 3C7 mAb was injected into the gland on days 4 and 10. Mice were killed under terminal anaesthesia on day 14 post cannulation. The submandibular glands were removed, with tissues partly snap-frozen in OCT (Sakura Finetek) for cryosectioning and partly stored in RNAlater (Ambion) for gene expression profiling.

### Bulk RNA-seq transcriptomics

RNA samples obtained from murine submandibular SGs were stored in RNAlater solution immediately after collection. Total RNA was extracted using the Qiagen RNeasy Mini Kit (Qiagen) according to the manufacturer’s instructions. RNA concentrations and purity were measured using a Qubit 2.0 Fluorometer (Invitrogen), and RNA integrity was assessed using the TapeStation system (Agilent Technologies). For each sample, 500 ng of total RNA was outsourced for bulk mRNA sequencing.

RNA samples were also obtained from labial SG lobules of patients with SjD, sourced from two distinct cohorts: an observational disease–control cohort consisting of patients with SjD and those with sicca as controls, and the TRACTISS randomized clinical trial, which compared rituximab to placebo with longitudinal data collected before and after treatment. Detailed patient information for the SG transcriptomic analysis cohorts is available^49^.

Labial SG biopsies were stored in RNAlater solution (Thermo Fisher Scientific) immediately following collection. Total RNA was extracted using the RNeasy Micro Kit (Qiagen) according to the manufacturer’s instructions. RNA concentrations and purity were quantified using a Qubit 2.0 Fluorometer (Invitrogen), and RNA integrity was assessed using the TapeStation system (Agilent Technologies). For each sample, 500 ng of total RNA was outsourced for bulk mRNA sequencing.

RNA-seq data were analysed using R (v.4.2.1) within the RStudio environment (v1.2.5042). Differentially expressed genes were identified using the DESeq2 package (v.4.2.1), comparing expression between the sicca and SjD groups, between placebo and rituximab groups in the TRACTISS trial cohort or between murine RNA-seq time points (0, 5 and 12 days post cannulation). The analysis focused solely on protein-coding genes. Gene expression levels were normalized using the variance stabilizing transformation method, and heatmaps were generated using the ComplexHeatmap package (v.2.6.2).

### Single-cell RNA-seq transcriptomics

Single-cell RNA-seq data generated from minor labial SG biopsies from patients with SjD or non-Sjögren’s sicca were accessed from the Gene Expression Omnibus database under accession code GSE272409. Data were analysed using R (v.4.4.0) and Seurat (v.5.1.0).

### qPCR analysis

Total RNA was extracted from the samples using the RNeasy Mini Kit (Qiagen) following the manufacturer’s protocol. The concentration and purity of the extracted RNA were assessed using a NanoDrop spectrophotometer (Thermo Fisher Scientific). Subsequently, 1 µg of RNA was retrotranscribed to complementary DNA (cDNA) using SuperScript IV Reverse Transcriptase (Invitrogen, Life Technologies) in accordance with the manufacturer’s instructions. The cDNA was diluted to a working concentration of 5 ng μl^−1^ and stored at −20 °C until further use. qPCR was conducted using the TaqMan PCR system. Each reaction contained 5 ng of cDNA per replicate, and all reactions were performed in duplicate. For gene expression analysis, the expression levels of both housekeeping and target genes were quantified simultaneously. This was achieved by incorporating a VIC-conjugated probe specific for 18S rRNA and FAM-conjugated probes for the target genes of interest. Relative gene expression levels were calculated using the ΔΔCt method. The list of probes used is provided in the Supplementary Data Table 7.

### Lactate quantification

Lactate concentrations were measured in the supernatant of SG organ cultures derived from multiple lobules from five patients with SjD with different degrees of histopathology focus scores, as well as in the submandibular SG tissue of both non-cannulated and cannulated mice, using the colourimetric l-Lactate Assay Kit (Abcam, ab65331).

For supernatant samples, after the completion of the organ culture, the supernatant was collected and stored at −80 °C until analysis. Lactate quantification was performed following the manufacturer’s instructions. In brief, standards were prepared using known concentrations of l-lactate and incubated with the provided reaction mix. The absorbance of the samples and standards was measured at 450 nm using a microplate reader (Synergy HT). Lactate concentrations were determined by comparing the absorbance values to the standard curve generated from known concentrations.

For SG tissue samples, tissues from non-cannulated and cannulated mice were collected, weighed and immediately homogenized in standard buffer using a tissue homogenizer. The homogenized tissue was then centrifuged, and the resulting supernatant was used for lactate quantification. Lactate levels in the tissue supernatants were measured using the same procedure as described for the organ culture supernatants.

### Immunofluorescence and immunohistochemical staining

Formalin-fixed, paraffin-embedded slides of SG biopsies from patients with SjD were selected based on the histological evidence of ELS^49^. Samples were classified as ELS+ (presence of ELS) or ELS− (absence of ELS). Tissue sections of 3 μm thickness were cut and mounted on polarized microscope slides. For hFFPE slides, deparaffinization was performed by immersion in xylene and rehydration through a series of graded ethanol solutions, followed by rinsing in distilled water. Heat-induced epitope retrieval was carried out using citrate buffer pH 6 (Agilent, S1699) at 95 °C for 30 min for staining combinations involving CD3 and SLC5A12, or using buffer pH 9 (Agilent, S2367) for staining combinations involving CD4 and SLC5A12. Following antigen retrieval, tissue sections were incubated in peroxidase blocking solution (Agilent) for 10 min to quench endogenous peroxidase activity, followed by protein blocking solution (Agilent) for 1 h at room temperature (22 ± 2 °C) to prevent non-specific binding. Primary antibodies were then added to the tissue sections and incubated for 1 h at room temperature. The details of the primary antibodies used are listed in Supplementary Data Table 8. After primary antibody incubation, slides were incubated with fluorochrome-conjugated secondary antibodies for 1 h at room temperature. Subsequently, slides were incubated with 5 μg ml^−1^ of 4′,6-diamidino-2-phenylindole (DAPI) in TBS (Life Technologies) for nuclear staining. Images were acquired using an Olympus IX81 fluorescence microscope with an Olympus digital camera (OrcaR-2). For the human images, whole-slide images were not acquired, and normalization to the total area was not applied. Instead, field images were specifically selected to include cell aggregates, either in the early stages of formation (initial stage-ELS) or fully organized (ELS+). Cell quantification was performed as the positive cell count per field.

For murine SG tissues, fresh-frozen murine SG tissues were used, with sections cut at 10 μm thickness using a cryostat. Hematoxilin and eosin (H&E) staining and direct double-immunofluorescence for CD3 and B220 were performed on sequential slides. The whole H&E slides were digitized using a Hamamatsu NanoZoomer S60 Slide Scanner at ×20 magnification. The digital analysis of the slides was conducted with the open-source software QuPath 0.6.0-rc3 (ref. ^56^).

For each H&E image, the digital imaging analysis included:

- Measurement of the total area of the gland, calculated using the software’s area calculation tools by semi-manually determining the perimeter of the SG tissue.
- Count of the number of inflammatory foci (peri-ductal leukocytic infiltrates with more than 50 lymphocytes).

The focus score was calculated as follows: focus score = (infiltrate number / total SG area (mm²)) × 4.

The image analysis of the immunofluorescence images was performed using cellSense software (Olympus). We identified regions on a pixel-by-pixel basis positive for DAPI and the markers of interest. To classify pixels as positive for DAPI and the markers of interest, we applied a global threshold on pixel intensities for the respective channels. The threshold values were assigned by a trained individual to ensure accurate identification of positively stained regions on all images. The global thresholds were implemented in QuPath^56^, and the annotations were then exported as ImageJ regions of interest^57^ and converted into simple features (sf library^58^) in the programming language R^59^. The area was quantified for regions in which markers of interest intersected the regions of positive pixels for the markers of interest. The areas were directly comparable between images, as the area of the images was fixed at 1,229,054.6934 µm². The areas were then compared, and a Wilcoxon statistical test was applied between the groups of interest. Aggregates segregation was evaluated by a trained individual, evaluating when CD3 and CD20 positive areas segregate in discrete areas.

The digital imaging analysis software was also used for the quantification of the aggregate area fraction on immunofluorescence images (CD3/B220) over the total area of the gland, measuring the fraction (%) of the total SG area occupied by the inflammatory aggregates. This complemented the information provided by the focus score, giving the dimension of the aggregates alongside the count provided by the focus score.

The area fraction of the aggregates was calculated as follows:

$$\begin{array}{l}{\rm{Aggregate}}\;{\rm{area}}\;{\rm{fraction}}\; ( \% )\\=({\rm{Aggregate}}\;{\rm{area}}\;({{\rm{mm}}}^{2})/{\rm{Total}}\;{\rm{SG}}\;{\rm{area}}\;({{\rm{mm}}}^{2}))\times 100\end{array}$$

For immunohistochemical staining of SLC5A12, human kidney tissue sections were first deparaffinized in xylene and rehydrated through a graded alcohol series. Antigen retrieval was performed by incubating the tissue sections in a citrate buffer (pH 6.0) and heating in a microwave for 10 min. After cooling, sections were washed in PBS and blocked with 5% normal goat serum for 1 h at room temperature to reduce non-specific binding. The primary antibody against SLC5A12 was applied to the tissue sections. The sections were incubated with the primary antibody overnight at 4 °C. After washing in PBS, sections were incubated with a secondary antibody conjugated to horseradish peroxidase for 1 h at room temperature. The staining signal was visualized using 3,3′-diaminobenzidine (DAB) as the chromogen, following the manufacturer’s protocol. Counterstaining was performed using haematoxylin for 2 min to visualize tissue morphology. Sections were then dehydrated, cleared in xylene and mounted with a coverslip using mounting medium.

### Acetylation array

CD4 T cells were isolated from mouse lymph nodes and spleen (Stemcell, 19852) and then activated and treated with lactate (10 mM) or left untreated over three time points: 12 h, 24 h and 48 h. Proteins were extracted with scioExtract buffer (Sciomics) using the extraction SOPs. After quality control of the samples, the bulk protein concentration was determined by bicinchoninic acid assay. The samples were labelled at an adjusted protein concentration for 2 h with scioDye 2. After 2 h, the reaction was stopped. Excess dye was removed and the buffer exchanged to PBS. All labelled protein samples were stored at −20 °C until use. The samples were analysed on scioDiscover antibody microarrays (Sciomics) targeting 1,352 different proteins with 1,821 antibodies. The arrays were blocked with scioBlock (Sciomics) on a Hybstation 4800 (Tecan). Subsequently, the samples were mixed with scioAcetyl detection mix, which enables detection of protein-specific acetylation, and incubated.

After incubation, the slides were thoroughly washed with 1× phosphate-buffered saline containing 0.05% Tween 20 (PBS-T), rinsed with 0.1 PBS-T and PBS, as well as with water, and subsequently dried with nitrogen. Slide scanning was conducted using a Powerscanner (Tecan) with constant instrument laser power and PMT settings. Spot segmentation was performed with GenePix Pro 6.0 (Molecular Devices). Acquired raw data were analysed using the linear models for microarray data (LIMMA) package of R-Bioconductor after uploading the median signal intensities. For normalization, a cyclic Loess normalization was applied. For analysis of the samples, a one-factorial linear model was fitted with LIMMA, resulting in a two-sided *t*-test or *F*-test based on moderated statistics. All presented *P* values were adjusted for multiple testing by controlling the false discovery rate according to Benjamini and Hochberg. Proteins were defined as differential for |log(fold change)| > 0.5 and an adjusted *P* < 0.05. Differences in protein abundance or acetylation level between different samples or sample groups are presented as log_2_(fold change).

### Western blot analysis

CD4 T cells were isolated from the spleen and lymph nodes of the mice. Cells were activated and subsequently treated with 10 mM lactate or left untreated for 24 h. After treatment, cells were collected and lysed in RIPA buffer (50 mM Tris-HCl pH 7.4, 150 mM NaCl, 1% NP-40, 0.5% sodium deoxycholate, 0.1% SDS) supplemented with protease and phosphatase inhibitors. The supernatants were collected, and protein concentration was determined using the BCA Protein Assay Kit (Pierce). Equal amounts of protein (30 µg per sample) were mixed with Laemmli sample buffer, boiled for 5 min, and separated by SDS–PAGE on a 10% polyacrylamide gel. The proteins were then transferred onto a PVDF membrane using a semi-dry transfer apparatus (Bio-Rad). Membranes were blocked with 5% non-fat dry milk in Tris-buffered saline with 0.1% Tween-20 (TBS-T) for 1 h at room temperature. The membranes were then incubated overnight at 4 °C with the following primary antibodies diluted in 5% BSA in TBS-T: Phospho-Stat3 (Tyr705) (Cell Signaling Technology, 1:1,000), Stat3 (Cell Signaling Technology, 1:1,000) and β-Actin (Cell Signaling Technology, 1:1,000). After washing three times with TBS-T, the membranes were incubated with horseradish peroxidase-conjugated secondary antibodies (anti-rabbit for Stat3 and pStat3, anti-mouse for β-Actin) (Cell Signaling Technology, 1:5,000) for 1 h at room temperature. The membranes were washed again three times with TBS-T, and the bound antibodies were detected using an enhanced chemiluminescence detection system (Thermo Scientific). The chemiluminescent signal was captured using a ChemiDoc Imaging System (Bio-Rad), and band intensities were quantified using ImageJ software (NIH). Data were analysed using GraphPad Prism software. Statistical significance was determined using a two-tailed Student’s *t*-test, with *P* < 0.05 considered statistically significant. Results are presented as means ± s.e.m. of at least three independent experiments.

### Cytokine measurements from ex vivo culture

PBMCs or SG (lip and parotid) biopsies, isolated from patients with SjD who had a positive lip biopsy for the histological evaluation for ELSs (confirmed by histology), were used for the in vitro culture. Each SG biopsy was cultured in RPMI 1640 medium supplemented with penicillin–streptomycin and 10% FBS for 24 h to allow for cell egression. Multiple samples from one MALT lymphoma parotidectomy and five SjD patient labial SG biopsies (2–8 lobules per patient) were tested. All culture supernatants were collected and frozen before cytokine analysis, as previously described. Cytokines were quantified using a customized multiplex liquid-phase immunoassay (Biolegend, LEGENDplex Human T_H_ Cytokine Panel), analysed with LEGENDplex data analysis software. A total of 12 human cytokines (IL-5, IL-13, IL-2, IL-6, IL-9, IL-10, IFNγ, TNF (TNFSF2), IL-17A, IL-17F, IL-4, IL-22) were simultaneously quantified. IL-21 levels were assessed with an ELISA kit (Biolegend, 433804) according to the manufacturer’s instructions. Cytokine production by human CD4^+^ and CD8^+^ cells (Granzyme-B, IFNγ, IL-17A and IL-21) was assessed by flow cytometry on egressed cells from SG organ culture following the addition of GolgiStop in the final 4 h of culture.

Murine SGs were collected from non-cannulated mice (controls) and Adv-cannulated mice at 5 and 12 days post cannulation. The samples were analysed for T cell phenotype and IL-21 cytokine production. At 4 h before tissue collection, mice were injected in the tail vein with 100 µl of 2.5 mg ml^−1^ brefeldin A resuspended in PBS to enhance intracellular cytokine detection. Murine SGs were enzymatically digested in medium containing collagenase D (Roche) and DNase I (Sigma-Aldrich) for 15 min at 37 °C to obtain single-cell suspensions.

Cells from both human and murine samples were stained for surface antigens, fixed, permeabilized (using fixation-permeabilization buffer; eBioscience) and stained for intracellular cytokines as previously described. The details of the antibodies used for flow cytometry are listed in Supplementary Data Table 9. Data acquisition was performed on an LSR Fortessa II flow cytometer (BD Biosciences) and analysed using FlowJo (v.10) software. BD Cytometer Setup and Tracking Beads were routinely used to calibrate the cytometer. Single-stain controls and fluorescence minus one controls were included to enable compensation and precise gating, respectively. For human experiments, events were gated on viable cells, while murine samples were gated on viable CD45^+^ cells. Where applicable, a dump channel was used to exclude non-relevant populations (V510/20 for human panels; R780/60 for murine panels).

The isolated PBMCs were cultured in RPMI 1640 medium supplemented with penicillin–streptomycin and 10% FBS. Cells were seeded in a 48-well plate at a density ranging from 200,000 to 300,000 cells per well. Cultures were maintained at 37 °C in a humidified atmosphere with 5% CO_2_. For activation, PBMCs were treated with 0.2 μg ml^−1^ soluble anti-CD3 (clone UCHT-1) and anti-CD28 (clone CD28.2) monoclonal antibodies, and pre-incubated for 1 h with anti-SLC5A12 monoclonal or its isotype control before stimulation with lactate (10 mM) for 24 h. Cytokine production, specifically IL-21, was measured in the culture supernatant using an IL-21 ELISA kit (Biolegend, 433804) according to the manufacturer’s instructions. IL-21 concentrations in the samples were determined by comparison to a standard curve generated using known concentrations of recombinant IL-21. Each sample was assayed in duplicate to ensure accuracy and reproducibility.

### Statistical analysis

All statistical analyses were performed using GraphPad Prism 10. Data are presented as means ± s.d. unless otherwise indicated. Normality of data distributions was assessed using the Shapiro–Wilk test. The specific statistical tests used, sample size and post hoc corrections, where applicable, are provided in the corresponding figure legends.

Sample sizes were determined based on preliminary experiments and effect sizes observed in prior studies. Investigators were blinded to group allocation during data collection and analysis when feasible. All statistical tests were two-tailed, and *P* < 0.05 was considered statistically significant. No data were excluded unless predefined criteria were met.

### Reporting summary

Further information on research design is available in the Nature Portfolio Reporting Summary linked to this article.

## Supplementary information

Supplementary Information Supplementary Tables 1–9.

Reporting Summary

## Data Availability

The RNA-seq data are publicly accessible via the following repositories and web interfaces: i) observational disease-control cohort (SjD and sicca): 10.6084/m9.figshare.29211473, and https://sjogren.hpc.qmul.ac.uk/; ii) TRACTISS randomized clinical trial: ArrayExpress under accession code E-MTAB-15225, and https://tractiss.hpc.qmul.ac.uk/. The single-cell RNA-seq data are publicly accessible via the Gene Expression Omnibus under accession code GSE272409. Software code created for this study can be obtained at https://gitlab.bham.ac.uk/spillfsystems-mechanobiology-health-disease/els-analysis. All other data are available in the main text or in the Extended Data.

## Code availability

Software code created for this study can be obtained at https://gitlab.bham.ac.uk/spillf-systems-mechanobiology-health-disease/els-analysis.
