# Supplementary material for: Lactate signalling leads to aggregation of immune-inflammatory hotspots and SLC5A12 blockade promotes their resolution
Source: Nat Metab. 2025 Aug 4;7(8):1663–80. doi: 10.1038/s42255-025-01331-9 (PMC12373510; doi:10.1038/s42255-025-01331-9)
Supplement: Supplementary file 1 — Supplementary Tables 1–9. [file 42255_2025_1331_MOESM1_ESM.pdf]

nature metabolism

Supplementary information https://doi.org/10.1038/s42255-025-01331-9

Lactate signalling leads to aggregation of immune-inflammatory hotspots and SLC5A12 blockade promotes their resolution

In the format provided by the authors and unedited

Supplementary Data Table 1. Proteins with differential abundance in untreated samples vs Lactate 12h samples. Proteins with a positive logFC value had a higher abundance in Lactate 12h samples, proteins with a negative value in untreated samples. In addition, p values adjusted for multiple testing are listed.

Protein	Uniprot-Entry	HGNC	logFC	AveExp	Adj. P-Val
**IL12B**	P29460	IL12B	1.15	12.57	3.5e-02
**HPT**	P00738	HP	-0.52	15.72	3.5e-02
**SPIT2**	O43291	SPINT2	-0.52	13.31	3.5e-02
**LEUK**	P16150	SPN	-0.54	11.96	4.3e-02
**IRF4**	Q15306	IRF4	-0.56	12.60	3.5e-02
**CCD50**	Q8IVM0	CCDC50	-0.59	11.15	3.9e-02
**ECHM**	P30084	ECHS1	-0.62	12.66	2.0e-02
**AMFR**	Q9UKV5	AMFR	-0.67	15.12	2.6e-03
**BID**	P55957	BID	-0.73	12.25	3.6e-03
**ANM5**	O14744	PRMT5	-0.82	11.44	9.3e-03
**CASP3**	P42574	CASP3	-1.05	13.35	7.0e-06

Supplementary Data Table 2. Proteins with differential abundance in untreated samples vs Lactate 24h samples. Proteins with a positive logFC value had a higher abundance in Lactate 24h samples, proteins with a negative value in untreated samples. In addition, p values adjusted for multiple testing are listed.

Protein	Uniprot-Entry	HGNC	logFC	AveExp	Adj. P-Val
**IL21**	Q9HBE4	IL21	2.57	10.02	2.1e-05
**G3P**	P04406	GAPDH	2.21	13.06	4.3e-02
**CADH5**	P33151	CDH5	2.17	12.30	1.5e-03
**GDN**	P07093	SERPINE2	2.17	12.98	4.2e-04
**CD14**	P08571	CD14	1.96	10.76	1.6e-02
**MUC1**	P15941	MUC1	1.68	10.17	2.6e-02
**MK01**	P28482	MAPK1	1.47	11.22	1.6e-02
**EDIL3**	O43854	EDIL3	1.40	12.58	2.0e-02
**BRPF3**	Q9ULD4	BRPF3	1.13	10.40	1.6e-02
**IL17**	Q16552	IL17A	1.07	12.25	1.6e-02
**CALX**	P27824	CANX	0.99	10.86	2.8e-02
**VEGF165+PLGFdimer**	P15692	VEGFA	0.88	10.09	1.6e-02
**SRGEF**	Q9UGK8	SERGEF	0.81	10.03	4.9e-02
**SF3B4**	Q15427	SF3B4	0.73	10.94	1.6e-02
**1433Z**	P63104	YWHAZ	0.67	9.69	1.6e-02
**TNFL8**	P32971	TNFSF8	0.63	8.67	1.6e-02
**EGF**	P01133	EGF	0.61	10.18	4.9e-02
**WIPF1**	O43516	WIPF1	0.52	6.83	2.9e-02
**P53**	P04637	TP53	-0.53	10.57	3.1e-02
**CYTL1**	Q9NRR1	CYTL1	-0.59	15.82	1.2e-02
**TSP1**	P07996	THBS1	-0.60	15.77	1.2e-02
**CCL5**	P13501	CCL5	-0.60	10.06	2.9e-02
**HPT**	P00738	HP	-0.66	15.72	1.2e-02
**CD70**	P32970	CD70	-0.71	9.44	2.9e-02
**ANM5**	O14744	PRMT5	-0.71	11.44	4.9e-02
**CYR61**	O00622	CYR61	-1.12	9.62	4.6e-02

Supplementary Data Table 3. Proteins with differential abundance in untreated samples vs Lactate 48h samples. Proteins with a positive logFC value had a higher abundance in Lactate 48h samples, proteins with a negative value in untreated samples. In addition, p values adjusted for multiple testing are listed.

Protein	Uniprot-Entry	HGNC	logFC	AveExp	Adj. P-Val
**CD14**	P08571	CD14	2.93	10.76	9.4e-06
**MUC1**	P15941	MUC1	2.08	10.17	3.3e-04
**CCL7**	P80098	CCL7	2.01	8.53	2.3e-02
**KI67**	P46013	MKI67	1.95	13.14	1.1e-02
**MSLN**	Q13421	MSLN	1.90	9.68	3.3e-03
**BDNF**	P23560	BDNF	1.79	12.31	3.9e-04
**1C01**	P30499	HLA-C	1.74	11.75	2.0e-05
**GDN**	P07093	SERPINE2	1.57	12.98	1.5e-03
**HMMR**	O75330	HMMR	1.54	10.25	1.5e-06
**ZDHC6**	Q9H6R6	ZDHHC6	1.44	11.73	2.0e-03
**CO5**	P01031	C5	1.44	8.76	3.7e-03
**EGLN**	P17813	ENG	1.36	9.71	3.7e-03
**OLR1**	P78380	OLR1	1.30	12.78	3.6e-02
**TNR11**	Q9Y6Q6	TNFRSF11A	1.23	13.64	3.8e-03
**CCL28**	Q9NRJ3	CCL28	1.20	11.47	4.0e-02
**GSTP1**	P09211	GSTP1	1.17	10.76	3.7e-03
**S10A9**	P06702	S100A9	1.12	9.85	6.3e-03
**POSTN**	Q15063	POSTN	1.11	13.96	4.5e-03
**ESR1**	P03372	ESR1	1.06	14.53	4.1e-02
**TNR9**	Q07011	TNFRSF9	1.01	11.56	4.2e-02
**FLT3**	P36888	FLT3	0.99	8.74	1.7e-02
**UXS1**	Q8NBZ7	UXS1	0.98	11.52	8.3e-03
**BARD1**	Q99728	BARD1	0.97	10.65	3.6e-03
**CAV2**	P51636	CAV2	0.96	9.89	4.1e-02
**APC**	P25054	APC	0.96	9.57	1.1e-03
**RLA0**	P05388	RPLP0	0.94	8.50	3.3e-03
**SF3B4**	Q15427	SF3B4	0.94	10.94	4.2e-05
**PARP1**	P09874	PARP1	0.92	13.25	3.7e-03
**LEG7**	P47929	LGALS7;LGALS7B	0.90	10.08	6.6e-05
**STAT1**	P42224	STAT1	0.89	11.64	4.2e-05
**TSN12**	O95859	TSPAN12	0.88	11.20	2.8e-02
**BRPF3**	Q9ULD4	BRPF3	0.88	10.40	1.4e-02
**TLR3**	O15455	TLR3	0.88	14.22	3.6e-03
**LRMP**	Q12912	LRMP	0.85	10.17	2.0e-03
**VEGFD**	O43915	VEGFD	0.83	8.72	3.2e-02
**LYAM2**	P16581	SELE	0.81	9.31	8.2e-03
**IL17**	Q16552	IL17A	0.81	12.25	1.2e-02

**ISK1**	P00995	SPINK1	0.80	10.65	2.5e-02
**IFNG**	P01579	IFNG	0.80	10.36	1.3e-03
**MUC2**	Q02817	MUC2	0.78	11.43	3.0e-03
**GRP**	P07492	GRP	0.76	12.07	2.0e-02
**GPM6B**	Q13491	GPM6B	0.76	8.53	2.3e-03
**CCL7**	P80098	CCL7	0.76	11.40	7.8e-04
**MIF**	P14174	MIF	0.73	9.57	2.9e-02
**CD20**	P11836	MS4A1	0.73	11.12	2.5e-02
**RL29**	P47914	RPL29	0.71	9.34	5.0e-04
**CATH**	P09668	CTSH	0.71	11.53	8.3e-03
**TFPI1**	P10646	TFPI	0.71	9.99	8.3e-03
**COMP**	P49747	COMP	0.71	10.63	1.0e-02
**HNRPF**	P52597	HNRNPF	0.70	10.37	3.7e-02
**RB**	P06400	RB1	0.69	10.04	7.6e-03
**OTUB1**	Q96FW1	OTUB1	0.69	10.44	3.3e-03
**1433Z**	P63104	YWHAZ	0.69	9.69	1.4e-03
**DPOLL**	Q9UGP5	POLL	0.68	11.15	7.6e-03
**MARK4**	Q96L34	MARK4	0.68	11.55	1.6e-02
**REQU**	Q92785	DPF2	0.68	10.38	4.5e-02
**IL6RA**	P08887	IL6R	0.67	12.04	6.6e-05
**PDCD2**	Q16342	PDCD2	0.66	11.06	6.8e-03
**RL18**	Q07020	RPL18	0.65	9.16	2.1e-03
**IL2**	P60568	IL2	0.64	9.68	4.5e-03
**LEUK**	P16150	SPN	0.63	11.95	1.4e-03
**FGF2**	P09038	FGF2	0.62	11.11	1.5e-03
**MYCN**	P04198	MYCN	0.62	11.28	2.0e-02
**CCD50**	Q8IVM0	CCDC50	0.61	11.15	5.9e-03
**EPCAM**	P16422	EPCAM	0.60	10.31	2.5e-03
**CCNB1**	P14635	CCNB1	0.60	9.07	2.9e-02
**LEG3**	P17931	LGALS3	0.60	9.23	2.7e-02
**TN13B**	Q9Y275	TNFSF13B	0.59	11.82	1.6e-05
**AURKB**	Q96GD4	AURKB	0.55	9.10	2.0e-02
**IGF1R**	P08069	IGF1R	0.54	10.32	1.7e-02
**IL13**	P35225	IL13	0.54	11.05	4.8e-02
**NP1L4**	Q99733	NAP1L4	0.53	9.51	8.1e-03
**TRML1**	Q86YW5	TREML1	0.53	9.55	4.0e-02
**JAK3**	P52333	JAK3	0.52	12.63	4.9e-02
**TNFL6**	P48023	FASLG	0.52	9.22	4.4e-02
**NCOR1**	O75376	NCOR1	0.50	11.52	4.4e-02
**FOLR2**	P14207	FOLR2	0.50	9.50	3.4e-02
**HPT**	P00738	HP	-0.55	15.72	3.6e-03
**DHX40**	Q8IX18	DHX40	-0.55	10.25	2.7e-02
**UBE2T**	Q9NPD8	UBE2T	-0.56	11.97	3.1e-02
**TCTP**	P13693	TPT1	-0.56	10.18	1.1e-02
**CD70**	P32970	CD70	-0.56	9.44	2.1e-02
**C3a anaphyl.**	P01024	C3	-0.57	10.14	3.0e-02
**RN141**	Q8WVD5	RNF141	-0.57	9.00	8.1e-03
**IGF2**	P01344	IGF2	-0.58	10.74	9.1e-03
**HPT**	P00738	HP	-0.59	8.75	2.7e-02
**PRTN3**	P24158	PRTN3	-0.59	9.25	3.4e-02
**IDS**	P22304	IDS	-0.60	10.84	4.8e-02

**SORL**	Q92673	SORL1	-0.60	10.93	1.5e-02
**HGFA**	Q04756	HGFAC	-0.61	10.20	2.0e-03
**IRF4**	Q15306	IRF4	-0.62	12.60	3.0e-03
**CD63**	P08962	CD63	-0.62	10.12	2.7e-02
**MELPH**	Q9BV36	MLPH	-0.63	9.79	1.8e-02
**GRN**	P28799	GRN	-0.65	12.54	1.5e-02
**CK5P3**	Q96JB5	CDK5RAP3	-0.66	10.69	3.3e-03
**S10A8/9**			-0.67	11.53	4.2e-03
**RET4**	P02753	RBP4	-0.68	11.68	2.4e-02
**RSSA**	P08865	RPSA	-0.68	10.34	2.0e-02
**SERC**	Q9Y617	PSAT1	-0.70	11.10	4.1e-02
**KAP2**	P13861	PRKAR2A	-0.72	10.06	4.5e-03
**IL12p70**	P29459	IL12A	-0.73	13.85	1.5e-02
**P2Y12**	Q9H244	P2RY12	-0.73	9.11	9.8e-03
**TNR14**	Q92956	TNFRSF14	-0.75	10.13	2.2e-04
**MRPP3**	O15091	KIAA0391	-0.75	9.61	1.3e-02
**CD81**	P60033	CD81	-0.76	9.57	2.7e-02
**FUT8**	Q9BYC5	FUT8	-0.76	11.02	7.1e-03
**CCND2**	P30279	CCND2	-0.76	13.10	5.5e-03
**UBP4**	Q13107	USP4	-0.76	11.14	1.9e-02
**PDCD1**	Q15116	PDCD1	-0.77	8.86	2.5e-02
**MMP11**	P24347	MMP11	-0.79	9.27	5.4e-03
**IBP7**	Q16270	IGFBP7	-0.79	10.44	4.9e-02
**IL6**	P05231	IL6	-0.79	11.68	5.3e-04
**TAF12**	Q16514	TAF12	-0.79	10.61	4.2e-02
**CADM1**	Q9BY67	CADM1	-0.80	10.05	7.4e-04
**ATRN**	O75882	ATRN	-0.81	10.40	7.4e-04
**CFAB**	P00751	CFB	-0.81	10.73	1.2e-02
**A1AT**	P01009	SERPINA1	-1.01	11.58	3.7e-03
**NCAM1**	P13591	NCAM1	-1.07	11.35	5.3e-04
**CD5**	P06127	CD5	-1.07	12.53	1.9e-02
**IL13 MOUSE**	P20109	Il13	-1.19	10.92	1.5e-03
**MICA**	Q29983	MICA	-1.31	12.35	4.2e-05
**EIF3I**	Q13347	EIF3I	-1.40	14.15	1.0e-02
**FINC**	P02751	FN1	-1.79	11.36	4.6e-02

Supplementary Data Table 4: Proteins with differential acetylation in untreated samples vs Lactate 12h samples. Proteins with a positive logFC value were more acetylated in Lactate 12h samples, proteins with a negative value in untreated samples. In addition, p values adjusted for multiple testing are listed.

Protein	Uniprot-Entry	HGNC	logFC	AveExp	Adj. P-Val
**STAT1**	P42224	STAT1	-0.68	12.75	7.5e-05
**SIGL5**	O15389	SIGLEC5	-0.89	9.17	1.4e-03

Supplementary Data Table 5: Proteins with differential acetylation in untreated samples vs Lactate 24h samples. Proteins with a positive logFC value were more acetylated in Lactate 24h samples, proteins with a negative value in untreated samples. In addition, p values adjusted for multiple testing are listed.

Protein	Uniprot-Entry	HGNC	logFC	AveExp	Adj. P-Val
**LYAM1**	P14151	SELL	0.63	10.43	4.9e-02
**CD99R**	P14209	CD99	0.56	11.89	4.6e-02
**IL6RA**	P08887	IL6R	-0.67	13.09	4.6e-02
**STAT1**	P42224	STAT1	-0.72	12.75	1.2e-04
**SIGL5**	O15389	SIGLEC5	-0.78	9.17	4.6e-02
**IL1B**	P01584	IL1B	-0.79	11.11	4.6e-02
**IL1B**	P01584	IL1B	-0.81	11.55	4.6e-02
**ZN593**	O00488	ZNF593	-0.91	11.39	4.6e-02
**C2C4B**	A6NLJ0	C2CD4B	-1.06	12.71	6.7e-05

Supplementary Data Table 6: Overview of RNA-seq cohorts

Cohort	Tissue	Time Point	Cohort Size	Sub-Groups
Observational Disease-Control	Lip salivary gland Biopsy	Baseline	93 patients	Sjögren: 51
				Sicca: 42
Randomized Clinical Trial	Lip salivary gland Biopsy	Week 0	21 patients	Placebo: 14
				RTX: 7
		Week 16	13 patients	Placebo: 7
				RTX: 6
		Week 48	12 patients	Placebo: 7
				RTX: 5

Supplemantary Data Table 7: TaqMan probes for real-time PCR

				Producer
AICDA	Hs00757808_m1	FAM	Human	Applied Biosystems
BCL6	Hs00277037_m1	FAM		
CCL19	Hs00171149_m1	FAM		
CXCL13	Hs00737930_m1	FAM		
CXCR5	Hs00173527_m1	FAM		
IL-17A	Hs00174383_m1	FAM		
IL-21	Hs00222327_m1	FAM		
STAT1	Hs01012966_m1	FAM		
STAT3	Hs01047380_m1	FAM		
IL-22	Hs01574154_m1	FAM		
IL-27	Hs00377366_m1	FAM		
LTB	Hs00242739_m1	FAM		
CCR7	Hs01013468_m1	FAM		
SLC5A12	Hs01054645_m1	FAM		
18s	Hs99999901_s1	VIC		
Cxcr5	mm00432086_m1	FAM	Murine	
Cxcl13	mm01208154_g1	FAM		
Ccr7	mm00432608_m1	FAM		
Ccl19	mm00839967_g1	FAM		
Il17	mm00439619_m1	FAM		
Ltb	mm00434774_g1	FAM		
Ltbr	mm00440235_m1	FAM		
Il21	mm00517640_m1	FAM		
Il21r	mm00600317_m1	FAM		
Hprt	mm01318743_m1	VIC		

Supplementary Data Table 8: Antibodies for immunofluorescent staining

Primary Antibody	Conjugation	Source	Catalogue	Dilution
CD3 (human)	None	Agilent	M7254	1:100
CD3 (mouse)	Alexa Fuor 488	Biolegend	100210	1:100
CD4 (human)	None	Agilent	M7310	1:100
CD20 (human)	None	Dako	M0755	1:400
B220 (mouse)	Alexa Fuor 647	Biolegend	103226	1:200
SLC5A12 (human)	None	Sigma-Aldrich	HPA045181	1:50

	Source		Dilution
Alexa Fluor 555 anti-mouse	Invitrogen	A-21121	1:300
Alexa Fluor 488 anti-rabbit	Invitrogen	A-21241	1:300

Summplementary Data Table 9: Antibodies for flowcytometry

**Human samples**							
**Antigen**	**Fluorochrome**	**Filter**	**Clone**	**Company**	**Cat. Numb**	**Batch**	**Ab volume in 100ul (ul)**
Surface:							
Zombie Viable Dye	Aqua	V525/50					
CD14	BV510	V525/50	M5E2	BioLegend	301842	B261483	2.5
CD19	BV510	V525/50	HIB19	BioLegend	302242	B221987	1
CD56	BV510	V525/50	HCD56	Biolegend	318340	B235441	2
CXCR5	BV605	V610/20	J252D4	Biolegend	356930	B242459	2.5
CD4	PE-Dazzle594	YG610/20	RPA-T4	Biolegend	300548	B245173	0.3
CD8	APC-Cy7	R780/60	SK1	Biolegend	344714	B240888	2.5
ICOS	PE-Cy7	YG780/60	C398.4A	Biolegend	313520	B155585	1
PD1	PerCP-Cy5	B695/40	EH12.2H7	Biolegend	329913	B238928	1.25
CD25	BV650	V660/20	BC96	Biolegend	302633	B227756	1.25
**Intracellular:**							
Granzyme B	Pacific Blue	V450/50	GB11	Biolegend	515407	B220715	5
IL-17A	BV711	V710/50	BL168	Biolegend	512328	B241946	5
INFg	BV785	V780/60	4S.B3	Biolegend	502542	B226924	5
IL-21	AF647	R670/14	3A3-N2	Biolegend	513006	B176680	2.5
Foxp3	PE	YG582/15	150D	Biolegend	320008	B246346	5

Murine samples							
**Antigen**	**Fluorochrome**	**Filter**	**Clone**	**Company**	**Cat. Numb**	**Batch**	**Ab volume in 100ul (ul)**
**Surface:**							
CD45	PerCp/Cy55	B695/40	30-F11	BioLegend	103131	B205158	0.12
B220	APC-Cy7	R780/60	RA3-6B2	BioLegend	103223	B202710	0.6
NK1.1	APC-Cy7	R780/60	PK136	BioLegend	108723	B197398	1
Gr1	APC-Cy7	R780/60	RB6-8C5	BioLegend	108423	B203034	0.2
F480	APC-Cy7	R780/60	BM8	BioLegend	123117	B202743	0.6
PD-1	BV785	V780/60	29F.1A12	Biolegend	135225	B210953	0.12
CXCR5	BV650	V660/20	L138D7	Biolegend	145517	B207123	2
CD25	BV605	V610/20	PC61	Biolegend	102035	B195571	0.6
CD4	PE/Cy7	YG780/60	GK1.5	Biolegend	100421	B196263	0.25
CD127	Pe-CF594	YG610/20	A7R34	Biolegend	135031	B204489	0.25
**Intracellular:**							
IL-17A	Alx488	B530/ 30	TC11-18H10.1	Biolegend	506909	B193887	0.25
IL-21	Alx647	R670/14	BL25168	Biolegend	516803	B201762	0.06
IFNg	PE	YG582/15	XMG1.2	Biolegend	505807	B194985	0.12
