# Supplementary material for: Lactate signalling leads to aggregation of immune-inflammatory hotspots and SLC5A12 blockade promotes their resolution
Source: Nat Metab. 2025 Aug 4;7(8):1663–80. doi: 10.1038/s42255-025-01331-9 (PMC12373510; doi:10.1038/s42255-025-01331-9)
Supplement: Supplementary file 2 — Reporting Summary [file 42255_2025_1331_MOESM2_ESM.pdf]

nature portfolio

Corresponding author(s): Michelangelo Certo, Michele Bombardieri, Claudio Mauro

Last updated by author(s): May 5, 2025

Reporting Summary

Nature Portfolio wishes to improve the reproducibility of the work that we publish. This form provides structure for consistency and transparency in reporting. For further information on Nature Portfolio policies, see our Editorial Policies and the Editorial Policy Checklist.

Statistics

For all statistical analyses, confirm that the following items are present in the figure legend, table legend, main text, or Methods section.

n/a	Confirmed	
☐	☒	The exact sample size (*n*) for each experimental group/condition, given as a discrete number and unit of measurement
☒	☐	A statement on whether measurements were taken from distinct samples or whether the same sample was measured repeatedly
☐	☒	The statistical test(s) used AND whether they are one- or two-sided<br>*Only common tests should be described solely by name; describe more complex techniques in the Methods section.*
☒	☐	A description of all covariates tested
☐	☒	A description of any assumptions or corrections, such as tests of normality and adjustment for multiple comparisons
☐	☒	A full description of the statistical parameters including central tendency (e.g. means) or other basic estimates (e.g. regression coefficient) AND variation (e.g. standard deviation) or associated estimates of uncertainty (e.g. confidence intervals)
☐	☒	For null hypothesis testing, the test statistic (e.g. *F*, *t*, *r*) with confidence intervals, effect sizes, degrees of freedom and *P* value noted<br>*Give P values as exact values whenever suitable.*
☒	☐	For Bayesian analysis, information on the choice of priors and Markov chain Monte Carlo settings
☒	☐	For hierarchical and complex designs, identification of the appropriate level for tests and full reporting of outcomes
☒	☐	Estimates of effect sizes (e.g. Cohen's *d*, Pearson's *r*), indicating how they were calculated

Our web collection on statistics for biologists contains articles on many of the points above.

Software and code

Policy information about availability of computer code

Data collection	Data collection was conducted without the use of software.
Data analysis	- CellSense software (Olympus)<br>- QuPath 0.6.0-rc3<br>- Graphpad Prism 10<br>- R (v.4.2.1) within RStudio environment (v1.2.5042)<br>- FlowJo V.10<br>- LEGENDplex™ Data Analysis Software

For manuscripts utilizing custom algorithms or software that are central to the research but not yet described in published literature, software must be made available to editors and reviewers. We strongly encourage code deposition in a community repository (e.g. GitHub). See the Nature Portfolio guidelines for submitting code & software for further information.

Data

Policy information about availability of data

All manuscripts must include a data availability statement. This statement should provide the following information, where applicable:

- Accession codes, unique identifiers, or web links for publicly available datasets
- A description of any restrictions on data availability
- For clinical datasets or third party data, please ensure that the statement adheres to our policy

The RNA-seq data are publicly accessible through the following web interfaces:
i) Observational disease-control cohort (Sjogren's disease and sicca): https://sjogren.hpc.qmul.ac.uk/
ii) TRACTISS randomized clinical trial: https://tractiss.hpc.qmul.ac.uk/
iii) Software code created for this study can be obtained at: https://gitlab.bham.ac.uk/spillfsystems-mechanobiology-health-disease/els-analysis
All other data are available in the main text or in the Extended Data.

Research involving human participants, their data, or biological material

Policy information about studies with human participants or human data. See also policy information about sex, gender (identity/presentation), and sexual orientation and race, ethnicity and racism.

Reporting on sex and gender

Information on sex and/or gender of the human participants has not been reported in the current manuscript. Sex of the QMUL cohort participants according to hospital notes is reported below (see population characteristics) and was previously reported for the TRACTISS trial in the original manuscripts (see population characteristics below)

Reporting on race, ethnicity, or other socially relevant groupings

Information on race and/or ethnicity of the human participants has not been reported in the current manuscript

Population characteristics

QMUL cohort characteristic are reported below

Sjogren (n=51) Sicca (n=42)
Sex F/M 43 /8 37 /5
TRACTISS trial general population characteristics are extensively reported in
Age in years
Mean [Median] (SD) 54.84 [55.00] 13.31 56.62 [56.50] 13.02
Disease duration in years Mean [Median] (SD) 7.20 [6.00] 6.19 4.65 [2.00] 5.34
ESSDAI at biopsy
Mean [Median] (SD) 5.89 [5.00] 4.94 /
Anti-Ro (Ro/SSA)
Positive of total (%) 32 /51 (62.75%) 0 /38 (0%)
Anti-La (La/SSB)
Positive of total (%) 21 /51 (41.18%) 0 /38 (0%)
Rheumatoid factor (RF)
Positive of total (%) 25 /47 (57.44%) 2 /37 (5.41%)
Anti-Nuclear antibody (ANA) Positive of total (%) 29 /37 (78.38%) 12 /41 (29.27%)
Serum IgG (g/L)
Mean [Median] (SD) 15.75 [13.85] 7.27 11.01 [11.20] 2.57
Serum IgA (g/L)
Mean [Median] (SD) 2.96 [2.51] 1.64 2.75 [2.30] 2.83
Serum IgM (g/L)
Mean [Median] (SD) 1.43 [1.18] 1.03 1.30 [1.06] 0.80
Serum C3 (g/L)
Mean [Median] (SD) 1.23 [1.20] 0.29 1.29 [1.32] 0.21
Serum C4 (g/L)
Mean [Median] (SD) 0.25 [0.25] 0.11 0.29 [0.27] 0.09
ELS Negative / Positive/ NA 23 / 24 / 4 /

TRACTISS total cohort characteristics are extensively reported in "Randomized Controlled Trial of Rituximab and Cost-Effectiveness Analysis in Treating Fatigue and Oral Dryness in Primary Sjogren's Syndrome.
Bowman SJ, Everett CC, O'Dwyer JL, Emery P, Pitzalis C, Ng WF, Pease CT, Price EJ, Sutcliffe N, Gendi NST, Hall FC, Ruddock SP, Fernandez C, Reynolds C, Hulme CT, Davies KA, Edwards CJ, Lanyon PC, Moots RJ, Roussou E, Giles IP, Sharples LD, Bombardieri M. Arthritis Rheumatol. 2017 Jul;69(7):1440-1450. doi: 10.1002/art.40093. Epub 2017 Jun 5"

TRACTISS salivary gland biopsy cohort characteristics are extensively reported in "Serum and Tissue Biomarkers Associated With Composite of Relevant Endpoints for Sjögren Syndrome (CRESS) and Sjögren Tool for Assessing Response (STAR) to B Cell-Targeted Therapy in the Trial of Anti-B Cell Therapy in Patients With Primary Sjögren Syndrome (TRACTISS).
Pontarini E, Sciacca E, Chowdhury F, Grigoriadou S, Rivellese F, Murray-Brown WJ, Lucchesi D, Fossati-Jimack L, Nerviani A, Jaworska E, Ghirardi GM, Giacomassi C, Emery P, Ng WF, Sutcliffe N, Everett C, Fernandez C, Tappuni A, Seror R, Mariette X, Porcher R, Cavallaro G, Pulvirenti A, Verstappen GM, de Wolff L, Arends S, Bootsma H, Lewis MJ, Pitzalis C, Bowman SJ, Bombardieri M; Trial for Anti–B Cell Therapy in Patients With Primary Sjögren Syndrome Study Research Group.

	Arthritis Rheumatol. 2024 May;76(5):763-776. doi: 10.1002/art.42772. Epub 2024 Feb 15"
Recruitment	The cross-sectional QMUL cohort consists of a monocentric cohort of consecutive patients with a diagnosis of Sjogren's disease or sicca/non specific chronic sialoadenitis who consented to donate labial salivary gland tissue for research obtained during diagnostic labial salivary gland biopsies.<br>The TRACTISS cohort consists of a multicentric UK cohort of patients with Sjogren's disease enrolled in the randomised double-blind placebo controlled TRACTISS trial. The TRACTISS trial design, with inclusion and exclusion criteria have been extensively presented in the original manuscript reporting the results of the trial in "Randomized Controlled Trial of Rituximab and Cost-Effectiveness Analysis in Treating Fatigue and Oral Dryness in Primary Sjogren's Syndrome.<br>Bowman SJ, Everett CC, O'Dwyer JL, Emery P, Pitzalis C, Ng WF, Pease CT, Price EJ, Sutcliffe N, Gendi NST, Hall FC, Ruddock SP, Fernandez C, Reynolds C, Hulme CT, Davies KA, Edwards CJ, Lanyon PC, Moots RJ, Roussou E, Giles IP, Sharples LD, Bombardieri M. Arthritis Rheumatol. 2017 Jul;69(7):1440-1450. doi: 10.1002/art.40093. Epub 2017 Jun 5"
Ethics oversight	The human study including disease-control cohort (sicca/Sjogren's disease) was approved by the Research Ethics Committee, reference 17/WS/0172 and 05/Q0702/1. The TRACTISS randomized clinical trial including the collection of biological samples was approved by Leeds Research Ethics Committee 10/H1307/99.

Note that full information on the approval of the study protocol must also be provided in the manuscript.

Field-specific reporting

Please select the one below that is the best fit for your research. If you are not sure, read the appropriate sections before making your selection.

☒ Life sciences ☐ Behavioural & social sciences ☐ Ecological, evolutionary & environmental sciences

For a reference copy of the document with all sections, see nature.com/documents/nr-reporting-summary-flat.pdf

Life sciences study design

All studies must disclose on these points even when the disclosure is negative.

Sample size	The QMUL cohort is a consecutive cross-sectional observational cohort of 51 Sjogren and 42 sicca patients undergoing diagnostic salivary gland biopsies who consented to donate additional tissue for research and for which sufficient material was obtained for bulk RNA sequencing. No formal power calculations were performed.<br>The TRACTISS trial sample size underwent formal power calculations as originally reported in "The TRACTISS protocol: a randomised double blind placebo controlled clinical trial of anti-B-cell therapy in patients with primary Sjögren's Syndrome. Brown S, Navarro Coy N, Pitzalis C, Emery P, Pavitt S, Gray J, Hulme C, Hall F, Busch R, Smith P, Dawson L, Bombardieri M, Wan-Fai N, Pease C, Price E, Sutcliffe N, Woods C, Ruddock S, Everett C, Reynolds C, Skinner E, Poveda-Gallego A, Rout J, Macleod I, Rauz S, Bowman S; TRACTISS trial team.<br>BMC Musculoskelet Disord. 2014 Jan 17;15:21. doi: 10.1186/1471-2474-15-21"
Data exclusions	Salivary gland biopsies for which either i) not sufficient RNA was available and/or ii) RNA quality as assessed by RIN or iii) did not pass quality control prior or after RNA sequencing were excluded from the study. The data presented from the cohort of 51 Sjogren and 42 sicca patients comprise patients who passed all the above quality control steps.
Replication	No bulk RNA sequencing data replication was available for this study.
Randomization	The TRACTISS trial involved randomization to either Rituximab or placebo as described in "The TRACTISS protocol: a randomised double blind placebo controlled clinical trial of anti-B-cell therapy in patients with primary Sjögren's Syndrome. Brown S, Navarro Coy N, Pitzalis C, Emery P, Pavitt S, Gray J, Hulme C, Hall F, Busch R, Smith P, Dawson L, Bombardieri M, Wan-Fai N, Pease C, Price E, Sutcliffe N, Woods C, Ruddock S, Everett C, Reynolds C, Skinner E, Poveda-Gallego A, Rout J, Macleod I, Rauz S, Bowman S; TRACTISS trial team.<br>BMC Musculoskelet Disord. 2014 Jan 17;15:21. doi: 10.1186/1471-2474-15-21"
Blinding	The TRACTISS trial is a double blinded trial as described in "The TRACTISS protocol: a randomised double blind placebo controlled clinical trial of anti-B-cell therapy in patients with primary Sjögren's Syndrome. Brown S, Navarro Coy N, Pitzalis C, Emery P, Pavitt S, Gray J, Hulme C, Hall F, Busch R, Smith P, Dawson L, Bombardieri M, Wan-Fai N, Pease C, Price E, Sutcliffe N, Woods C, Ruddock S, Everett C, Reynolds C, Skinner E, Poveda-Gallego A, Rout J, Macleod I, Rauz S, Bowman S; TRACTISS trial team. BMC Musculoskelet Disord. 2014 Jan 17;15:21. doi: 10.1186/1471-2474-15-21"

Reporting for specific materials, systems and methods

We require information from authors about some types of materials, experimental systems and methods used in many studies. Here, indicate whether each material, system or method listed is relevant to your study. If you are not sure if a list item applies to your research, read the appropriate section before selecting a response.

Materials & experimental systems

n/a	Involved in the study
[ ]	[x] Antibodies
[x]	[ ] Eukaryotic cell lines
[x]	[ ] Palaeontology and archaeology
[ ]	[x] Animals and other organisms
[ ]	[x] Clinical data
[x]	[ ] Dual use research of concern
[x]	[ ] Plants

Methods

n/a	Involved in the study
[x]	[ ] ChIP-seq
[ ]	[x] Flow cytometry
[x]	[ ] MRI-based neuroimaging

Antibodies

Antibodies used

Antibodies for histology

Primary Antibody	Conjugation	Source	Catalogue	Clone	Dilution
CD3 (human)	None	Agilent-Dako	M7254	F7.2.38	1:100
CD3 (mouse)	Alexa Fuor 488	Biolegend	100210	17A2	1:100
CD4 (human)	None	Agilent-Dako	M7310	4B12	1:100
CD20 (human)	None	Agilent-Dako	M0755	L26	1:400
B220 (mouse)	Alexa Fuor 647	Biolegend	103226	RA3-6B2	1:200
SLC5A12 (human)	None	Sigma-Aldrich	HPA045181	polyclonal	1:50

Antibodies for flowcytometry

Antibodies for flowcytometry staining for human samples

Antigen	Fluorochrome	Filter	Clone	Company	Cat. Numb	Batch	Working dilution (ul)
Surface:							
Zombie Viable Dye	Aqua	V525/50					
CD14	BV510	V525/50	M5E2	BioLegend	301842	B261483	2.5
CD19	BV510	V525/50	HIB19	BioLegend	302242	B221987	1
CD56	BV510	V525/50	HCD56	Biolegend	318340	B235441	2
CXCR5	BV605	V610/20	J252D4	Biolegend	356930	B242459	2.5
CD4	PE-Dazzle594	YG610/20	RPA-T4	Biolegend	300548	B245173	0.3
CD8	APC-Cy7	R780/60	SK1	Biolegend	344714	B240888	2.5
ICOS	PE-Cy7	YG780/60	C398.4A	Biolegend	313520	B155585	1
PD1	PerCP-Cy5	B695/40	EH12.2H7	Biolegend	329913	B238928	1.25
CD25	BV650	V660/20	BC96	Biolegend	302633	B227756	1.25
Intracellular:							
Granzyme B	Pacific Blue	V450/50	GB11	Biolegend	515407	B220715	5
IL-17A	BV711	V710/50	BL168	Biolegend	512328	B241946	5
INFg	BV785	V780/60	4S.B3	Biolegend	502542	B226924	5
IL-21	AF647	R670/14	3A3-N2	Biolegend	513006	B176680	2.5
Foxp3	PE	YG582/15	150D	Biolegend	320008	B246346	5

Antibodies for flowcytometry staining for murine samples

Antigen	Fluorochrome	Filter	Clone	Company	Cat. Numb	Batch	Working dilution (ul)
Surface:							
CD45	PerCp/Cy55	B695/40	30-F11	BioLegend	103131	B205158	0.12
B220	APC-Cy7	R780/60	RA3-6B2	BioLegend	103223	B202710	0.6
NK1.1	APC-Cy7	R780/60	PK136	BioLegend	108723	B197398	1
Gr1	APC-Cy7	R780/60	RB6-8C5	BioLegend	108423	B203034	0.2
F480	APC-Cy7	R780/60	BM8	BioLegend	123117	B202743	0.6
PD-1	BV785	V780/60	29F.1A12	Biolegend	135225	B210953	0.12
CXCR5	BV650	V660/20	L138D7	Biolegend	145517	B207123	2
CD25	BV605	V610/20	PC61	Biolegend	102035	B195571	0.6
CD4	PE/Cy7	YG780/60	GK1.5	Biolegend	100421	B196263	0.25
CD127	Pe-CF594	YG610/20	A7R34	Biolegend	135031	B204489	0.25
Intracellular:							
IL-17A	Alx488	B530/ 30	TC11-18H10.1	Biolegend	506909	B193887	0.25
IL-21	Alx647	R670/14	BL25168	Biolegend	516803	B201762	0.06
IFNg	PE	YG582/15	XMG1.2	Biolegend	505807	B194985	0.12

Validation

We validated the antibodies following a two-step approach to ensure specificity and optimal performance. First, we adhered closely to the manufacturer's guidelines regarding recommended dilutions and storage conditions. In addition, prior to conducting the actual experiments, we performed preliminary optimization assays to determine the most effective antibody dilutions under our specific experimental conditions. This included titration series and assessment of signal specificity and background. These steps allowed us to confidently proceed with the experiments using validated working concentrations.

Animals and other research organisms

Policy information about studies involving animals; ARRIVE guidelines recommended for reporting animal research, and Sex and Gender in Research

Laboratory animals	C57BL/6 mice (Charles River, UK) and Slc5a12 KO mice (produced by the Sanger Institute via CRISPR/Cas9). 10-13 week-old.
Wild animals	The study did not involve wild animals.
Reporting on sex	Yes, we are reporting the sex of the animals used in our study. For this research, we exclusively used male mice. The decision to use only male mice was based on minimizing biological variability introduced by hormonal fluctuations in females, which can affect immune responses and metabolic pathways relevant to this study. This approach ensured greater consistency and reproducibility in the experimental data.
Field-collected samples	Mice were housed in a controlled environment with a 12-hour light/dark cycle, with lights on at 7:00 a.m. and off at 7:00 p.m. They had ad libitum access to food and water and were kept at a constant temperature of 22 ± 2°C and humidity of 50 ± 10%.
Ethics oversight	The study was approved by the institutional Animal Welfare and Ethical Review Body (AWERB) and conducted in accordance with national and international guidelines for the care and use of laboratory animals. All experimental protocols were reviewed and approved to ensure adherence to ethical standards aimed at minimizing harm and distress to the animals. Animal housing, handling, and experimental procedures complied with the guidelines established under the Animal (Scientific Procedures) Act 1986 in the UK. Every effort was made to ensure the highest standards of welfare throughout the study.

Note that full information on the approval of the study protocol must also be provided in the manuscript.

Clinical data

Policy information about clinical studies

All manuscripts should comply with the ICMJE guidelines for publication of clinical research and a completed CONSORT checklist must be included with all submissions.

Clinical trial registration	TRACTISS (ISRCTN: 65360827, EudraCT Number: 2010-021430-64
Study protocol	The TRACTISS protocol: a randomised double blind placebo controlled clinical trial of anti-B-cell therapy in patients with primary Sjögren's Syndrome. Brown S, Navarro Coy N, Pitzalis C, Emery P, Pavitt S, Gray J, Hulme C, Hall F, Busch R, Smith P, Dawson L, Bombardieri M, Wan-Fai N, Pease C, Price E, Sutcliffe N, Woods C, Ruddock S, Everett C, Reynolds C, Skinner E, Poveda-Gallego A, Rout J, Macleod I, Rauz S, Bowman S; TRACTISS trial team. BMC Musculoskelet Disord. 2014 Jan 17;15:21. doi: 10.1186/1471-2474-15-21
Data collection	Clinical data were collected between August 2011and January 2014
Outcomes	TRACTISS results were reported in full in "Randomized Controlled Trial of Rituximab and Cost-Effectiveness Analysis in Treating Fatigue and Oral Dryness in Primary Sjogren's Syndrome. Bowman SJ, Everett CC, O'Dwyer JL, Emery P, Pitzalis C, Ng WF, Pease CT, Price EJ, Sutcliffe N, Gendi NST, Hall FC, Ruddock SP, Fernandez C, Reynolds C, Hulme CT, Davies KA, Edwards CJ, Lanyon PC, Moots RJ, Roussou E, Giles IP, Sharples LD, Bombardieri M. Arthritis Rheumatol. 2017 Jul;69(7):1440-1450. doi: 10.1002/art.40093. Epub 2017 Jun 5"

Plants

Seed stocks	N/A
Novel plant genotypes	N/A
Authentication	N/A

Flow Cytometry

Plots

Confirm that:

☒ The axis labels state the marker and fluorochrome used (e.g. CD4-FITC).

☒ The axis scales are clearly visible. Include numbers along axes only for bottom left plot of group (a 'group' is an analysis of identical markers).

☒ All plots are contour plots with outliers or pseudocolor plots.

☒ A numerical value for number of cells or percentage (with statistics) is provided.

Methodology

Sample preparation	Mononuclear cell suspensions were obtained from whole blood and salivary gland biopsies (lip and parotid) of patients with Sjögren's disease. Blood samples were processed using density gradient separation, while biopsies were placed in culture medium to allow spontaneous egression (24h) of cells.<br>For murine salivary glands samples, four hours prior to surgery, mice were injected via the tail vein with 100 µL of 2.5 mg/mL Brefeldin A (resuspended in PBS) to enhance intracellular cytokine staining. Murine salivary glands were collected and enzymatically digested in medium containing collagenase D (Roche) and DNase I (Sigma-Aldrich) for 15 minutes at 37°C to obtain a single-cell suspension.
Instrument	LSR Fortessa II (BD Biosciences) flow cytometer
Software	FlowJo V.10 software
Cell population abundance	N/A
Gating strategy	For human experiments, events were gated on viable cells, while murine samples were gated for viable/CD45+ cells. Where relevant, a dump channel was used to exclude nonrelevant populations (human panels V510/20, murine panels R780/60). Fluorescence minus one (FMO) controls were included to enable compensation and precise gating, respectively.

☒ Tick this box to confirm that a figure exemplifying the gating strategy is provided in the Supplementary Information.
